# Multi‐Omics and ‐Organ Insights into Energy Metabolic Adaptations in Early Sepsis Onset

**DOI:** 10.1002/advs.202504418

**Published:** 2025-05-24

**Authors:** Lin‐Lin Xu, Zhengyuan Zhou, Sascha Schäuble, Wolfgang Vivas, Karen Dlubatz, Michael Bauer, Sebastian Weis, Mervyn Singer, Roman Lukaszewski, Gianni Panagiotou

**Affiliations:** ^1^ Microbiome Dynamics Leibniz Institute for Natural Product Research and Infection Biology– Hans Knöll Institute 07745 Jena Germany; ^2^ Department of Anesthesiology and Intensive Care Medicine Jena University Hospital 07747 Jena Germany; ^3^ Bloomsbury Institute of Intensive Care Medicine Division of Medicine University College London London NW1 2BU UK; ^4^ Translational Infection Medicine Leibniz Institute for Natural Product Research and Infection Biology– Hans Knöll Institute 07745 Jena Germany; ^5^ Institute of Infectious Disease and Infection Control Jena University Hospital 07747 Jena Germany; ^6^ Faculty of Biological Sciences Friedrich Schiller University 07743 Jena Germany; ^7^ Jena University Hospital Friedrich Schiller University 07747 Jena Germany; ^8^ Department of Medicine University of Hong Kong Hong Kong SAR China; ^9^ Center for Sepsis Control and Care Jena University Hospital 07747 jena Germany

**Keywords:** cell–cell communication, energy imbalance, lipid metabolism, metabolic modeling, sepsis biomarkers, single‐nucleus RNA sequencing (snRNA‐Seq)

## Abstract

Systemic metabolic dysregulation in sepsis critically impacts patient survival. To better understand its onset, untargeted serum metabolomics and lipidomics are analyzed from 152 presymptomatic patients undergoing major elective surgery, and identified key metabolites, including serine and aminoadipic acid, that differentiate postoperative uncomplicated infection from sepsis. Using single‐nucleus RNA sequencing data from an in vivo mouse model of sepsis, tissue‐independent down‐regulation and tissue‐specific differences of serine and energy‐related genes including key module roles for the mitochondria‐linked genes, *Cox4i1*, *Cox8a*, and *Ndufa4* are identified. Finally, serine‐dependent metabolic shifts, especially in the liver, are revealed by using ^12^C/^13^C murine data with labeled serine, and link altered activity of the serine hydroxymethyltransferase (SHMT) cycle with perturbed purine metabolism during sepsis. This study demonstrates the close interrelationship between early metabolite changes and mitochondrial dysfunction in sepsis, improves the understanding of the underlying pathophysiology, and highlights metabolic targets to prospectively treat presymptomatic, but at‐risk, patients.

## Graphical abstract

This study shows that patients at risk of sepsis have a distinct metabolite and lipid signature, including serine and aminoadipic acid, in their serum before clinical diagnosis. A mouse model of sepsis with a compatible serum signature reveals underlying metabolic changes, including mitochondrial adaptation, altered serine‐dependent purine metabolism, and key gene factors as potential drug targets.

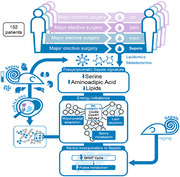

## 1 Introduction

Sepsis, defined as a life‐threatening organ dysfunction caused by a dysregulated response to infection,^[^ ^1^ ^]^ is a critical medical condition with a significant global impact, responsible for an estimated 48.9 million cases and 11 million deaths annually.^[^ ^2^ ^]^ Prompt recognition and treatment are key to improving survival,^[^ ^3^ ^]^ however early detection remains a substantial challenge, highlighting an urgent need for reliable early biomarkers.

Despite positive effects on survival by antimicrobial treatments (also prophylactic) and supportive care for failing organs, patient‐unspecific treatment protocols and the initial sepsis research focusing on managing inflammation yielded so far insufficient therapeutic success.^[^ ^3^, ^4^ ^]^ Over the last decade it became increasingly clear, that the failed metabolic adaptation disrupting the homeostatic capacity of the infected host is a central phenomenon that characterizes sepsis. Alongside, metabolic profiling has proposed biomarkers with the potential for clinical application as rapid diagnostic and prognostic tools in patients with sepsis.^[^ ^5^, ^6^, ^7^ ^]^ Improving our understanding of the early metabolic disturbances in sepsis appears therefore pivotal in our combat against its dire consequences.^[^ ^8^, ^9^, ^10^, ^11^ ^]^ Metabolic and energy‐associated changes including mitochondrial dysfunction^[^ ^12^, ^13^, ^14^, ^15^ ^]^ play a critical role in sepsis. These changes profoundly affect both immune and non‐immune cells and lead to dysfunction in multiple organs such as the heart, lungs, kidneys, liver, and brain. However, the vast majority of published clinical studies that have investigated the metabolic component of sepsis have been conducted on patients who are already critically ill.^[^ ^16^, ^17^ ^]^ Very few have been performed at an early stage^[^ ^18^ ^]^ or been targeted at metabolic disturbances such as hypoglycemia,^[^ ^19^, ^20^ ^]^ which may explain the limited success of interventions targeting identified metabolic failures. Early metabolic alterations, particularly those involving energy metabolism, may serve as adaptive processes beneficial to the host.^[^ ^11^, ^21^, ^22^ ^]^ Recognizing and harnessing these adaptive changes in patients at risk of developing sepsis hold the potential for novel preventive or therapeutic strategies to improve, interrupt or prevent disease courses and fatal patient outcomes.

Another understudied key aspect, besides the lack of appropriate human studies for identification of early metabolic adaptations in patients at risk of developing sepsis, is the intra‐ and inter‐tissue communication in disease. These are potential causes of the multi‐organ failure, of the energy‐associated pathways, and the dissipation of involved metabolites across organs.^[^ ^12^, ^16^, ^22^ ^]^ Creating a detailed multi‐tissue map that highlights cross‐tissue interactions from integrated data is essential for advancing our understanding and treatment (including prophylactically) of the metabolic and energetic maladaptation at the onset or during the course of early sepsis.

**Figure 1:**
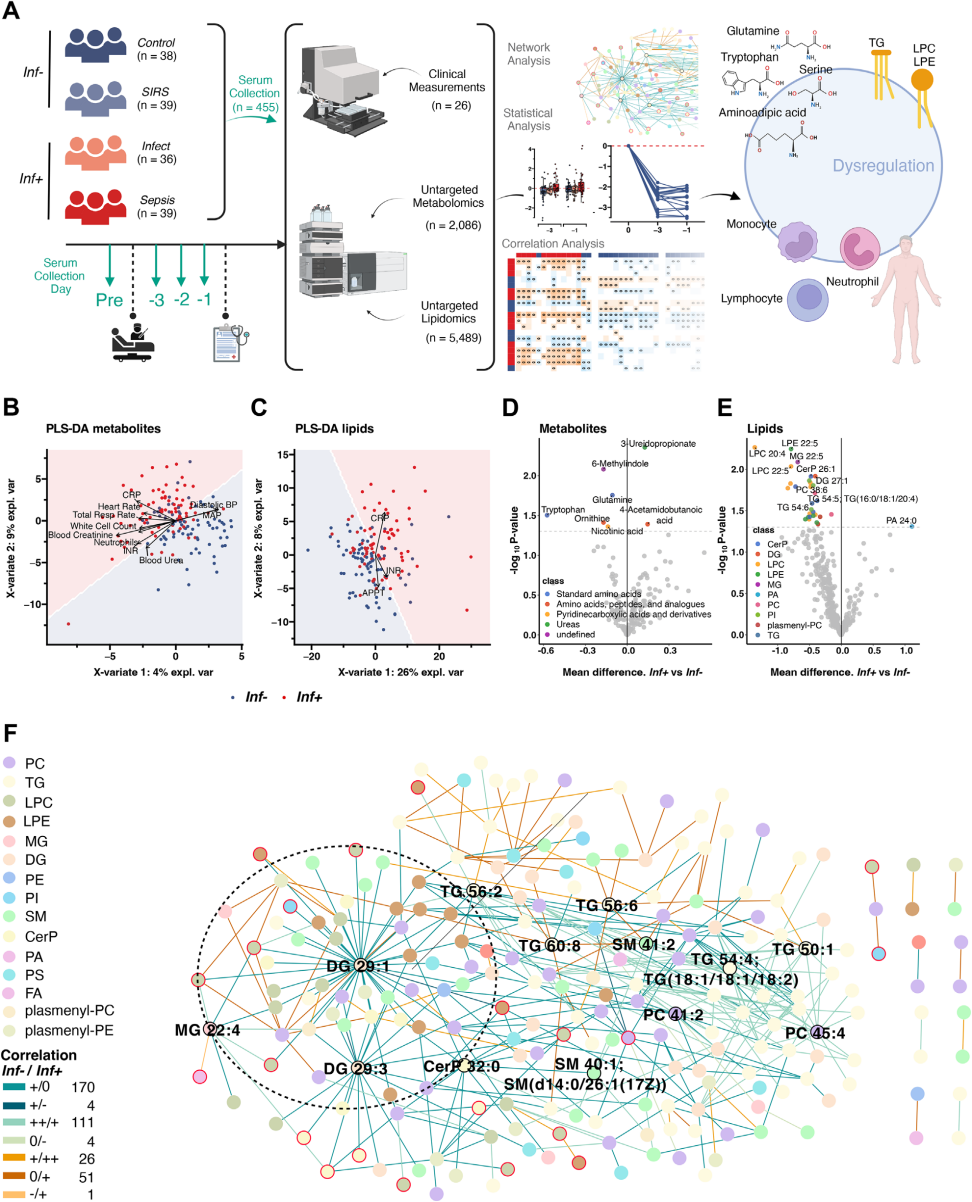
Infection‐specific metabolomics and lipidomics using presymptomatic serum samples from elective surgery patients. A) Study design. Serum samples were retrieved from 75 patients preoperatively (Pre) and 3 to 1 day(s) before clinical diagnosis of postoperative infection (39 developing *Sepsis* and 36 with uncomplicated *Infect* status). The patients were matched with 77 patients with no postoperative infection (38 *Control* and 39 *SIRS*). In total 455 samples were processed for untargeted metabolomics (n = 2086) and lipidomics (n = 5489) together with 26 tracked clinical parameters. *Infect*: uncomplicated postoperative infection; *SIRS*: systemic inflammatory response. Created with Biorender.com. B,C) Visualization of metabolomics/lipidomics data following Partial Least‐Squares Discriminant Analysis (PLS‐DA). Clinical parameters are indicated by individual arrows. *Inf+/Inf‐*: Patients with/without postoperative infection. D,E) Volcano plot of logistic regression models with covariates age, sex, and organ dysfunction status built separately with each metabolite (D) or lipid (E) to differentiate the groups infection (*Inf+*) from no infection (*Inf‐*). Metabolites and lipids with raw P‐value ≤ 0.05 are named and colored according to their HMDB and Lipid Map subclass. F) Multiscale embedded correlation network analysis illustrating the differential correlation of lipids and changes in lipid metabolic pathways in presymptomatic samples associated to *Inf‐* versus *Inf+* postoperative outcome. Only lipid pairs with significant differential correlations (adjusted empirical p ≤ 0.01) were included. Sign/sign in the legend indicates the direction and strength of correlation change in *Inf‐*/*Inf+* followed by the number of associated significant correlation changes. For instance, “++/+ 111″ indicates 111 lipid pairs correlate significantly stronger positive (++) in *Inf‐* than in *Inf+* (+). One module of biological interest with Diglyceride (DG) 29:1 as hub was circled for better visual clarity (see Results for details). Hub nodes of further modules (not shown) are named and with a black border. Significant lipids shown in (E) are indicated by a red border. APPT: activated partial thromboplastin time; SOFA: Sequential Organ Failure Assessment score; BP: blood pressure; GCS: Glasgow Coma Scale; INR: international normalized ratio; MAP: mean arterial pressure; Total Resp Rate: total respiratory rate; CRP: C‐Reactive Protein. TG: triglyceride; LPC: lysophosphatidylcholine; LPE: lysophosphatidylethanolamine; PC: phosphatidylcholine; Cerp: ceramide phosphate; SM: sphingomyeline; PE: phosphatidylethanolamine; DG: diglyceride; PI: phosphatidylinositol; DGDG: digalactosyldiacylglycerol; PA: phosphatidic acid; PS: phosphatidylserine; MG: monoacylglycerol; FA: fatty acid.

In order to fill current gaps in our knowledge, we performed multi‐time point, untargeted metabolomic, and lipidomic analyses of a unique cohort of patient serum samples taken before the onset of sepsis. We included a comparison against several control groups, thereby capturing early metabolic changes that may serve adaptive roles (**Figure** **1**A). Furthermore, we extended our research to a mouse model of sepsis and performed untargeted metabolomic and lipidomic analyses in serum and in five organs. With this additional data, we elucidate tissue‐tissue communication connected to the metabolic and lipidomic signature derived from our human cohort. To explore metabolic changes at the tissue and cell type level for potential conserved and cell‐specific responses, we performed single‐nucleus RNA sequencing (snRNA‐Seq) coupled with genome‐scale metabolic modeling across various tissues. After examining cell‐cell communication within bioenergetic pathways, we finally generated ^13^C/^12^C labeled serine data of a mouse sepsis model to further describe shifted metabolic fluxes and highlight a serine‐associated mechanistic metabolic shift across tissues and cells in sepsis. This data elucidates the location of systemic failure in sepsis, can be linked to metabolic differences present already in the serum of presymptomatic patients, and allows to explore new prophylactic and precision medicine measures to treat sepsis before its confirmed diagnosis.

## 2 Results

### 2.1 Clinical and Molecular Characterization of the Patient Cohort

A biobank with blood samples collected daily from 4385 patients undergoing elective surgery was established.^[^ ^23^, ^24^ ^]^ Demographic and clinical data were recorded commencing preoperatively and continuing for up to a week after, to enable identification of presymptomatic patients developing an infection, complicated or not by new‐onset organ dysfunction (sepsis). To capture the dynamic metabolic response of the host to infection and/or sepsis we retrieved serum samples from our biobank corresponding to time points *Pre* (pre‐operation), and postoperatively on days ‐3, ‐2, and ‐1 before the clinical diagnosis of infection or sepsis (Figure 1A). A total of 455 serum samples were retrieved from 152 patients (Figure 1A; Figure S1A and Table S1, Supporting Information), including 231 samples from 77 non‐infected (*Inf‐*) and 224 from 75 infected patients (*Inf+*). These groups were further discriminated by 114 samples from 38 non‐infected patients (*Inf‐*) making an uncomplicated postoperative recovery (*Control*), 117 samples from 39 patients developing a non‐infectious systemic inflammatory response (*SIRS*), 107 samples from 36 patients diagnosed with uncomplicated infection (*Infect*), and 117 samples from 39 patients diagnosed with *Sepsis* (Table S1, Supporting Information). In our subsequent analysis, we refer to presymptomatic patient samples by their respective postoperative outcome.

All samples were analyzed by liquid chromatography‐mass spectrometry (LC‐MS) for untargeted metabolomic and lipidomic characterization. After filtering initially detected 2086 metabolites and 5489 lipids, we continued further analysis with robustly identified 173 metabolites and 359 lipids across the different patient groups (see Experimental Section for details).

There was no significant difference in age (Kruskal‐Wallis rank sum test, *p* = 0.7) and sex (Pearson's Chi‐squared test, *p* = 0.6) between *Control*, *SIRS*, *Infect*, and *Sepsis* patient groups (Table S1, Supporting Information). Significant differences in blood urea, total respiratory rate, and Sequential Organ Failure Assessment (SOFA) score^[^ ^25^ ^]^ were found among the four patient groups, with the highest values recorded in sepsis patients (Table S1, Supporting Information, Kruskal–Wallis rank sum test, *p* ≤ 0.05). Notably, 8 out of 26 clinical characteristics of the *SIRS* group were more similar to the infected groups (including both *Sepsis* and *Infect*, Table S1, Supporting Information) than the *Control* group.

### 2.2 Metabolomics and Lipidomic Profiles Distinguish Infected Patients Before Clinical Identification

To identify early host metabolic responses to infection, we compared *Inf‐* and *Inf+* presymptomatic metabolomic and lipidomic profiles after normalizing postoperative by patient‐resolved preoperative metabolite and lipid abundances (see Experimental Section for details). Metabolite and lipid profiles distinguished (partial least‐squares discriminant analysis (PLS‐DA)) non‐infected from infected patients with an area under the curve (AUC) of 0.82 and a 95% confidence interval (CI) of 0.76–0.88 for metabolites and AUC of 0.79 (CI: 0.71–0.86) for lipids (Figure S1B,C, Supporting Information). Several clinical indices were significantly associated with observed variation in the metabolomic and lipidomic profiles based on partial least‐squares discriminant analysis (PLS‐DA, false discovery rate (FDR)‐adjusted *p* ≤ 0.05). Based on the metabolomic profiles, blood creatinine and respiratory rate were significantly associated with infection (Figure 1B). For both lipidomics and metabolomics, C‐reactive protein (CRP) was significantly associated with infection (Figure 1B,C). The top most influential analytes as identified by multivariable analysis included the metabolites 3−ureidopropionate, tryptophan, and glutamine, and the lipids lysophosphatidylcholine (LPC) 20:4, lysophosphatidylethanolamine (LPE) 22:5, and monoacylglycerol (MG) 22:5, respectively (Figure S1D,E, Supporting Information).

Early response biomarkers of infection were then sought by comparing presymptomatic *Inf‐* and *Inf+* patient groups for significantly different metabolite and lipid abundances (Figure 1D,E). Logistic regression models with age, sex, and organ dysfunction (observed in *Sepsis* and *SIRS* patients) as covariates (Figure 1D,E) identified significantly differing levels of seven metabolites (logistic regression, *p* ≤ 0.05, Cohen's d: 0.33 – 0.47) (Table S2, Supporting Information) and 30 lipids (logistic regression, *p* ≤ 0.05, FDR ≤ 0.4, Cohen's d: 0.33 – 0.51) (Table S2, Supporting Information). Among these, 3‐ureidopropionate, which acts as an endogenous neurotoxin via inhibition of mitochondrial energy metabolism,^[^ ^26^ ^]^ was elevated in *Inf+* (Figure 1D). Tryptophan and nicotinic acid both had lower values in *Inf+*, which may relate to insufficient cellular nicotinamide adenine dinucleotide (NAD^+^) supply and possible energy substrate deficiency.^[^ ^27^ ^]^

Of note, 29 of the 30 identified lipids from several classes were significantly decreased in *Inf+* (Figure 1E), while only PA 24:0 (phosphatidic acid) was increased, hinting at potential impairments in lipid and/or energy metabolism. To systematically evaluate perturbed lipid co‐regulation at the early presymptomatic stages of infection, a multiscale embedded correlation analysis was performed (Figure 1F). Overall, the direction and/or strength of 367 correlation pairs changed between *Inf‐* and *Inf+*, suggesting significant alterations in the host's lipid metabolism. 285 positive associations were reduced or lost in the early stages of infection compared to the non‐infection group, with 170 indicating a loss of positive correlation (+/0, blue lines), four indicating a switch from positive to negative correlation (±, dark blue lines) and 111 indicating a significant reduction in positive correlation (++/+, light blue lines, see Experimental Section for details) based on the difference in z‐scores between *Inf‐* and *Inf+* (Figure 1F).

From the global networks, six lipid modules were identified via multiscale clustering analysis (Table S3, Supporting Information). One of these (Figure 1F, encircled) included a cluster hub that connected to several significantly differing abundant lipids upon infection. This module comprises DG (diglyceride) 29:1 as a hub connected to numerous lipids including lysophosphatidylcholines (LPCs), lysophosphatidylethanolamines (LPEs), phosphatidylcholines (PCs), ceramide phosphates (Cerps) and sphingomyelines (SMs). Enrichment analysis showed that next to lysoglycerophospholipids and polyunsaturated fatty acids, this module enriches for “lipid−mediated signaling”, “endoplasmic reticulum” (ER), and “mitochondrion” (FDR ≤ 0.05), which suggests a regulatory influence of lipids involved in ER and mitochondria cell wall integrity (Figure S1F, Supporting Information).

In summary, our untargeted metabolomics and lipidomics analysis revealed distinct presymptomatic metabolic signatures that differentiate infected from non‐infected patients, with notable alterations in metabolites and lipid levels potentially linked to early host responses to infection.

### 2.3 Specific Amino Acids and Lipid Classes Changes Indicate Transition from Uncomplicated Infection to Sepsis

**Figure 2:**
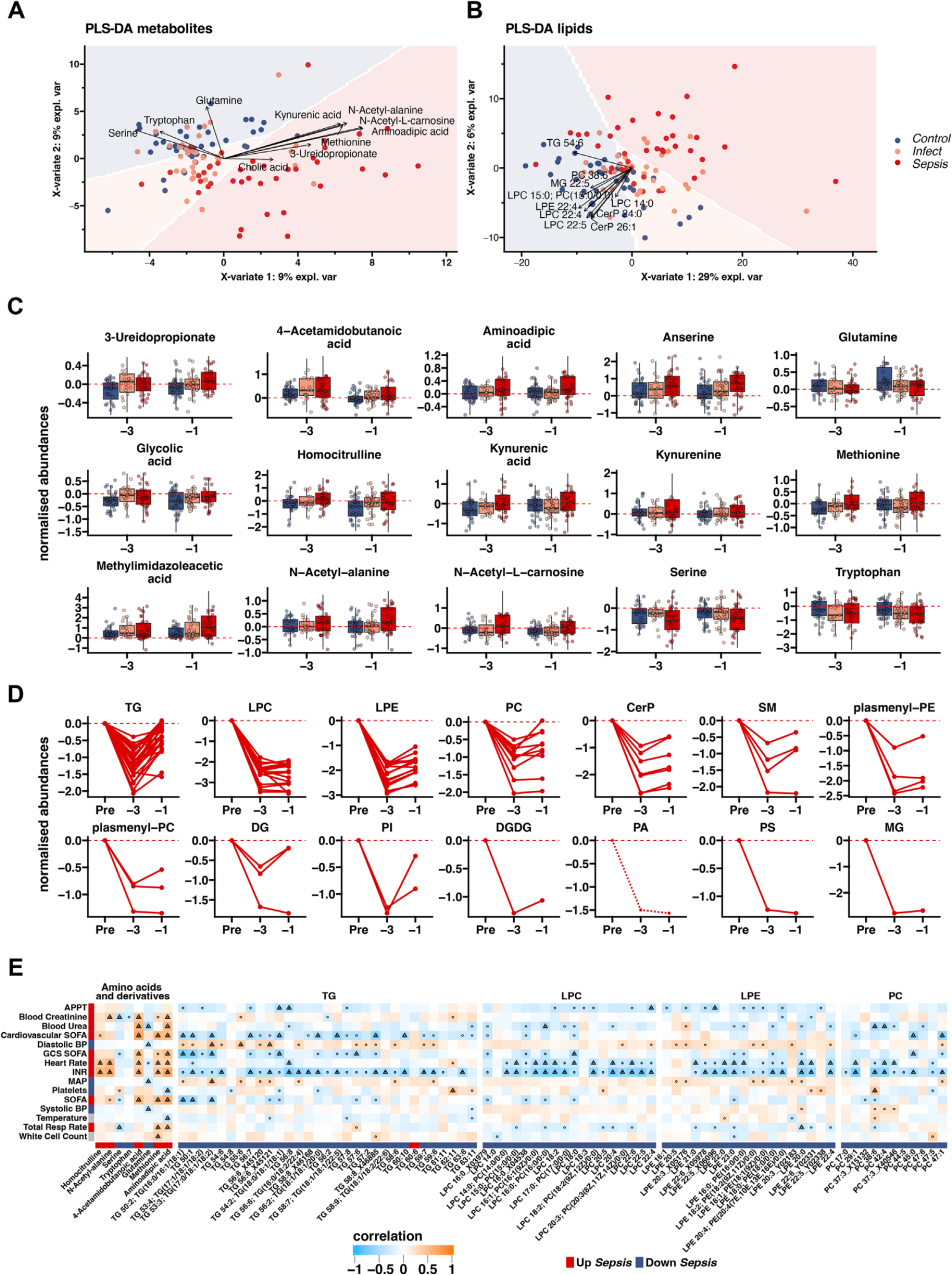
Presymptomatic severity trend analysis from Control to septic postoperative outcome. A,B) Visualization of PLS‐DA using metabolite (A) or lipid (B) data with different postoperative outcome. The top 10 most influential metabolites and lipids are indicated by individual arrows. C,D) Ordinal regression models with covariates age and sex for each metabolite and lipid with unadjusted p‐value ≤ 0.05 and false discovery rate (FDR) ≤ 0.25 of the disease severity trends from *Control* over *Infect* (uncomplicated infection) to *Sepsis* within 3 days and 1 day before diagnosis. Displayed abundances are normalized to preoperation abundances. (C) Significant metabolites in the *Control* (blue), *Infect* (orange), and *Sepsis* (red) group. D) Significant lipids in the *Sepsis* group were grouped by their name and present the change from preoperation to 1 day before (postoperative) diagnosis. Solid and dashed lines indicate higher and lower abundances (normalized by preoperative abundance) in the *Control* compared to the *Sepsis* group, respectively (compare Figure S2). E) Partial Spearman correlation analysis of significantly altered compounds from Figures (C), (D) together with clinical parameters in the *Sepsis* group. Significance of correlation is indicated by black circles (un‐adjusted P ≤ 0.05) and triangles (FDR ≤ 0.05). Annotation bars indicate increasing (red, Up *Sepsis*) and decreasing (blue, Down *Sepsis*) severity of infection as tested by ordinal regression models (compounds) and based on literature review (clinical data). APPT: activated partial thromboplastin time; SOFA: Sequential Organ Failure Assessment score; BP: blood pressure; GCS: Glasgow Coma Scale; INR: international normalized ratio; MAP: mean arterial pressure; Total Resp Rate: total respiratory rate. TG: triglyceride; LPC: lysophosphatidylcholine; LPE: lysophosphatidylethanolamine; PC: phosphatidylcholine; Cerp: ceramide phosphate; SM: sphingomyeline; PE: phosphatidylethanolamine; DG:.

We next explored, whether early host metabolite or lipid biomarkers can be related to infection outcome. Accordingly, a comparative analysis was performed of the presymptomatic untargeted metabolomic and lipidomic profiles from the *Control*, *Infect*, and *Sepsis* groups with PLS‐DA (**Figure** **2**A,B). Patients from *Control* (metabolite: AUC = 0.79, CI = 0.71–0.87; lipid: AUC = 0.85, CI = 0.79–0.92; Figure S2A, Supporting Information) and *Sepsis* (metabolite: AUC = 0.88, CI = 0.81–0.95; lipid: AUC = 0.83, CI = 0.75–0.91; Figure S2A, Supporting Information) groups could be well differentiated. However, the *Infect* group with uncomplicated infection outcome remained challenging to clearly separate from the other two groups (metabolite: AUC = 0.62, CI = 0.51–0.72; lipid: AUC = 0.6, CI = 0.49–0.71; Figure S2A, Supporting Information). The overall difference in data variance between groups (projection analysis, FDR‐adjusted *p* ≤ 0.05, see Experimental Section for details) was mainly driven by *Control‐*associated lipid classes LPCs, LPEs and triglycerides (TGs) as well as serine, and *Sepsis*‐associated aminoadipic acid (Figure 2A,B; Figure S2B, Supporting Information).

Ordinal regression models (adjusted for age and sex) were built using the presymptomatic profiles to identify early changes in the metabolites and lipids associated with increased disease severity (Figure 2C,D; Table S2, Supporting Information). The concentrations of 15 metabolites significantly differed between *Control*, *Infect*, and *Sepsis* (*p* ≤ 0.05, FDR ≤ 0.25). Methionine levels were lower in the *Control* group compared to the infection groups *Infect* and *Sepsis*, particularly three days before actual diagnosis (Figure 2C). In contrast, the concentration of the essential amino acids serine and glutamine decreased with increasing infection severity (Figure 2C). These are associated with support of the citric acid (TCA) cycle, maintaining redox balance and mitochondrial function.^[^ ^28^ ^]^ Serine is also recently described as an important regulator of lipid homeostasis with clear molecular links with sphingolipid metabolism.^[^ ^29^ ^]^ Aminoadipic acid was also found elevated in *Sepsis* compared to *Infect* and *Control* (Figure 2C). Spearman's correlation analysis showed strong and significant correlations (partial Spearman, adjusted for age and sex, *p* ≤ 0.05, FDR ≤ 0.05) between serine, methionine, and aminoadipic acid with several clinical parameters (Figure 2E). While serine was negatively correlated, methionine and aminoadipic acid were positively correlated with organ dysfunction (SOFA score, Figure 2E), suggesting an involvement of these amino acids in the transition from simple infection to sepsis.

In contrast to metabolites, more lipids were significantly changed in abundance between *Control*, *Infect*, and *Sepsis* groups (N = 106; *p* ≤ 0.05, FDR ≤ 0.25) (Figure 2D). Even though a recent study suggested an association between lipid metabolism and mitochondrial dysfunction as a predictor of COVID‐19 prognosis,^[^ ^30^ ^]^ it remains unclear whether a disrupted lipid homeostasis is cause or consequence of mitochondrial dysfunction in various pathophysiological conditions. We grouped significantly altered lipids according to their class and their abundance changes from the preoperative level to one day before diagnosis of sepsis (Figure 2D). All significantly altered lipids decreased toward the day of diagnosis in the *Sepsis* group. The abundance decrease was greater for *Sepsis* compared to *Control* and *Infect* except for two lipids (PA 24:0, TG (triglycerides) 61:11) (Figure S2C, Supporting Information).

The most altered lipid group was triglycerides (TGs), however, the prognostic or even therapeutic value of TGs in sepsis is controversial.^[^ ^31^, ^32^ ^]^ In our study, 30 out of 31 significantly altered TGs decreased with increasing infection severity in presymptomatic diagnosis of sepsis patients (Figure S2C, Supporting Information). However, TG abundances increased in one day compared to three days before diagnosis of sepsis with 25 of 30 TGs recovering more than half of their initially reduced abundance (Figure S2C, Supporting Information), which suggests time‐sensitive TG dynamics at the onset of sepsis. Likewise, TG 50:1, TG 50:2, and TG 53:3, which were significantly negatively correlated with the SOFA score (FDR ≤ 0.05) (Figure 2E), were found to first decrease and then increase before diagnosis. Of the other lipid classes, LPCs (n = 18), LPEs (n = 18) and PCs (n = 11) showed a significant and stable decrease with infection severity (Figure 2D). Most of the significantly altered lipids showed statistically significant negative correlations (partial Spearman, adjusted for age and sex, *p* ≤ 0.05 and FDR ≤ 0.05) with the SOFA score and other clinical parameters (Figure S2D, Supporting Information). Selected lipids with the strongest correlations were LPC 16:0 (r = −0.42), LPE 23:0 (r = −0.38), and PC 37:3 (r = −0.33).

We additionally explored whether *Sepsis* induces different early metabolic and lipidomic changes than *SIRS* by Spearman correlation analysis including also clinical indices (302500 pairwise comparisons in total, Table S4, Supporting Information). After detection of five mega‐clusters (Figure S3, Supporting Information), we superimposed the *Sepsis*‐based cluster hierarchy on *SIRS* correlations. Next to three clusters with moderate similarity (Spearman's r = 0.52. r = 0.57, r = 0.57, respectively), two clusters showed lower similarity (r = 0.35, r = 0.44, respectively) between *Sepsis* and *SIRS*. These later two included metabolites associated to TCA cycle, serine, and threonine metabolism, as well as several lipid pathways, including mitochondrion and membrane components, and several phospholipids, which are associated with mitochondrial integrity and function^[^ ^33^ ^]^ (Figure S3, Supporting Information).

Overall, we observed pronounced changes in the levels of specific amino acids and derivatives, as well as glycerophospholipids (LPCs, LPEs, and PCs) that correlate with organ dysfunction (indicated by a change in SOFA scores) in the *Sepsis* group. These all point toward metabolic and particularly mitochondrial dysfunction. Notably, among the altered metabolites, serine was the only amino acid being significantly negatively correlated with SOFA scores. Despite these findings, the underlying mechanisms of organ dysfunction remain unexplored due to the infeasibility of collecting prospective organ data in patient cohorts scheduled for operation and having different postoperative complications.

### 2.4 Differential Tissue Responses and Inter‐Tissue Communication in a CLP Mouse Model of Sepsis

After having identified human presymptomatic signatures of sepsis involving lipid, mitochondrial, and amino acid metabolism and due to the lack of organ data of human patients, we sought to further explore the signature's relevance for systemic metabolic dysfunction in a murine model of sepsis (Figure S4A, Supporting Information). The survival rate 24 h after surgery is similar between septic (cecal ligation and puncture, CLP) and control (sham) mice (Figure S4B, Supporting Information). However, septic mice show reduced body weight, food intake, temperature, and glucose levels, along with an increased disease score compared to control mice (Figure S4C, Supporting Information). Septic mice also exhibit significantly reduced total activity during both day and night (Figure S4D, Supporting Information). Most immune cell populations and pathogen loads are similar between septic and control mice (Figure S4E,F, Supporting Information), with the exception of elevated monocytes in the bone marrow and increased neutrophils and myeloid cells in the primary lymphoid follicle of septic mice.

**Figure 3:**
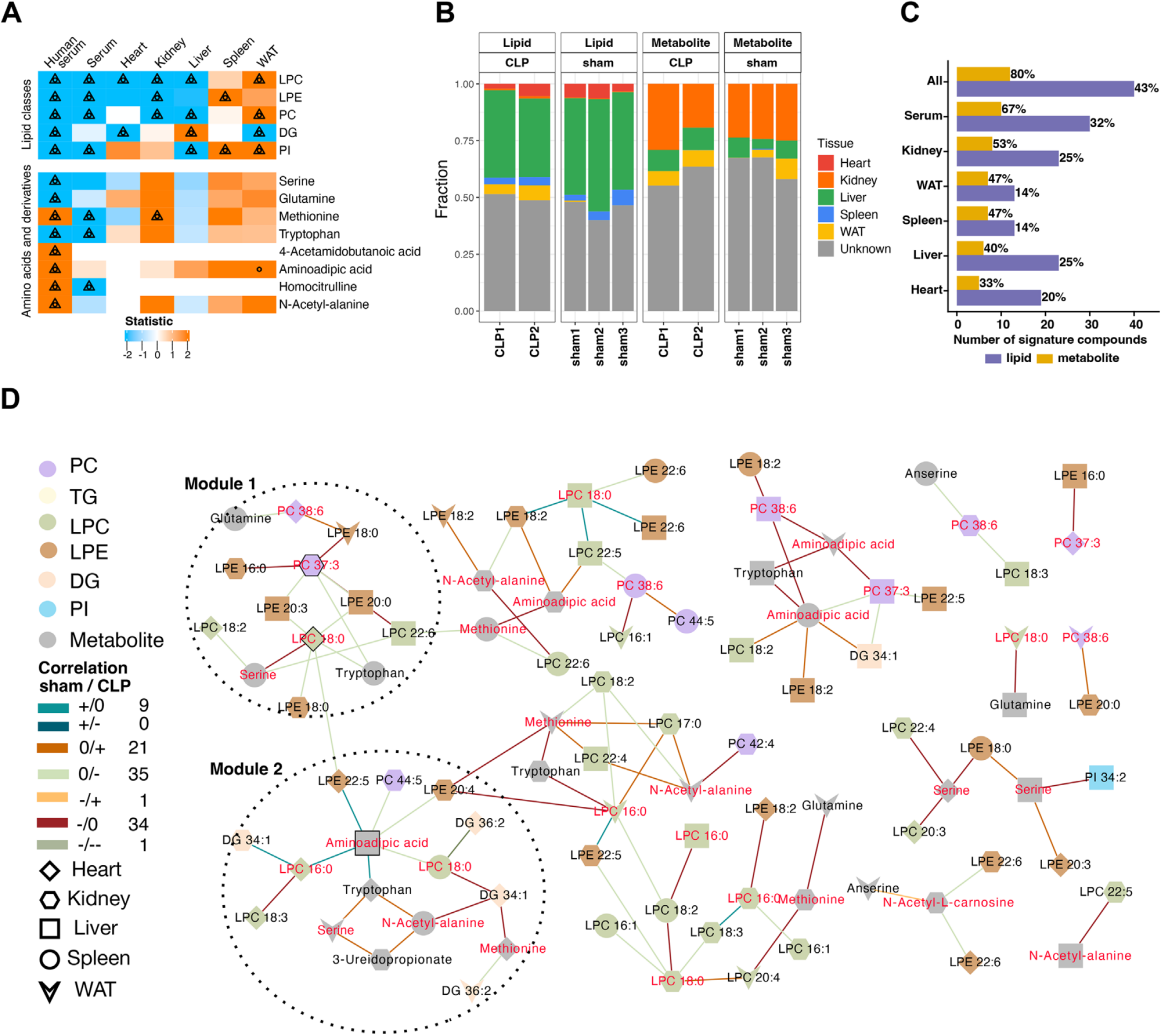
A CLP‐induced septic mouse model recaptures human sepsis signature compounds. A) Statistical analysis for CLP versus sham mice samples of metabolites and lipids associated to *Sepsis* signature compounds identified in the presymptomatic human cohort. Significance according to two‐tailed Student's t‐test (circle: unadjusted p‐value ≤ 0.05, triangle: FDR ≤ 0.25). Significance of human serum samples (Figure 2C,D) is additionally indicated for easier comparison. B) Fast expectation‐maximization microbial source tracking (FEAST) results show the estimated contribution of five tissues (“source”) on the serum (“sink”) based on all metabolites and lipids composition for each mouse (two septic, CLP1/2, and three control, sham1/2/3, mice). C) Absolute number and fraction of human sepsis signature compounds also detected in mice serum and five tissues. D) Multiscale embedded correlation network analysis illustrating the differential correlation of metabolites and lipids in sham relative to CLP. Only correlation pairs with significant differential correlations (adjusted empirical P ≤ 0.05) were included. Sign/sign indicates the direction and strength of correlation changes in sham/CLP followed by the number of associated lipid pairs with this change. Two identified modules are circled for clarity (compare Results). Module hub nodes have a black border, while nodes with red labels are compounds that significantly correlated with SOFA score in the human cohort (Figure S2B). Abbreviations: LPC: lysophosphatidylcholine; LPE: lysophosphatidylethanolamine; PC: phosphatidylcholine; DG: diglyceride; PI: phosphatidylinositol; TG: triglyceride; WAT: white adipose tissue.

We performed untargeted metabolomic and lipidomic analyses in mouse serum and in addition, in five organ tissues, including heart, liver, kidney, spleen, and white adipose tissue (WAT) 24 h after operation. Several lipid groups (LPCs, LPEs, PCs, DGs, and phosphatidylinositols (PIs)), and, with the exception of methionine, amino acids and their derivatives (serine, glutamine, tryptophan, and aminoadipic acid) that were significantly altered in the serum of human presymptomatic *Sepsis* patients, were also altered in the same direction in the serum of septic CLP mice (**Figure** **3**A). At the tissue level, different tissues showed different abundance changes in these metabolites and lipids (Figure 3A; Table S2, Supporting Information). Lipid groups, including LPCs, LPEs, and PCs, showed a decreasing trend in heart, kidney, and liver tissues (Figure 3A). Specifically, LPCs decreased significantly (*p* ≤ 0.05 and FDR ≤ 0.25; Cohen's d in [−0.61, −1.19]) in all three tissues, PCs decreased significantly (*p* ≤ 0.05 and FDR ≤ 0.25) in liver (Cohen's d = −0.46) and kidney (Cohen's d = −0.64), while LPEs decreased significantly (*p* ≤ 0.05, FDR ≤ 0.25, Cohen's d = −1.05) in kidney (Figure 3A). In contrast, spleen and WAT exhibited an increasing trend for these lipids (Figure 3A). Notably, LPCs (Cohen's d = 1.24) and PCs (Cohen's d = 0.97) increased significantly (*p* ≤ 0.05, FDR ≤ 0.05) in WAT, while LPEs increased significantly (*p* ≤ 0.05, FDR ≤ 0.05, Cohen's d = 1.01) in spleen (Figure 3A).

To further understand the contribution of the five tissues to serum metabolites and lipids, we adapted the Fast expectation‐maximization microbial source tracking (FEAST) algorithm^[^ ^34^ ^]^ (Figure 3B). Across all mice, the largest contribution to serum lipids was attributed to the liver (mean 42%), while the kidney was the largest contributor to metabolites (mean 24%). On average, 53% of serum lipids and 38% of serum metabolites could be attributed to the studied tissue sources (Figure 3B). Further high percentages of the serum lipids (47%) and metabolites (62%) were attributed to unknown sources (Figure 3B), which may be due to the close relationship between serum metabolites and the gut microbiome.^[^ ^35^ ^]^ By cross‐checking against the Microbial Metabolites Database (MiMeDB),^[^ ^36^ ^]^ we indeed found that 86% of the metabolites and 47% of the lipids could be derived from the gut microbiome.

Of the 121 metabolites and lipids identified in our human study as presymptomatic sepsis signatures, 80% of the metabolites and 43% of the lipids were detected in the serum or five other tissues in mice with mouse serum showing the highest overlap (metabolite: 67%; lipid: 32%, Figure 3C). Multiscale embedded correlation analysis revealed that the direction or strength of 101 correlation pairs involving human presymptomatic sepsis signature metabolites and lipids changed between the mouse sepsis and control groups, indicating a substantial alteration in inter‐tissue communication (Figure 3D). Notably, two modules were identified from the global network using multiscale clustering analysis (Figure 3D, encircled). Module 1 includes PC 37:3 from the kidney and LPC 18:0 from the heart as module hubs (Figure 3D). LPC 18:0, linked to serine and tryptophan in the spleen, has been shown to have therapeutic effects in experimental sepsis.^[^ ^37^ ^]^ Aminoadipic acid from the liver is the hub of module 2, connected to multiple compounds correlated significantly with the SOFA score in the human cohort, including LPC 16:0 in heart and LPC 18:0 in spleen (Figure 3D). Three further affected amino acids or derivatives in this module across different tissues were serine, methionine, and N‐acetyl‐alanine (Figure 3D).

Overall, we also observed the early changes of important metabolic and lipid classes, we identified in the serum of the presymptomatic human cohort, in mouse serum. However, a distinct metabolic regulation in different mouse tissues was also notable, suggesting a potential rewiring of energy metabolic pathways in a tissue‐specific manner. These findings point to the possibility that transcriptional changes before or during early sepsis may underlie the observed metabolic shifts.

### 2.5 Genome‐Scale Modeling Identifies Cellular Drivers of Metabolic Changes in Sepsis

**Figure 4:**
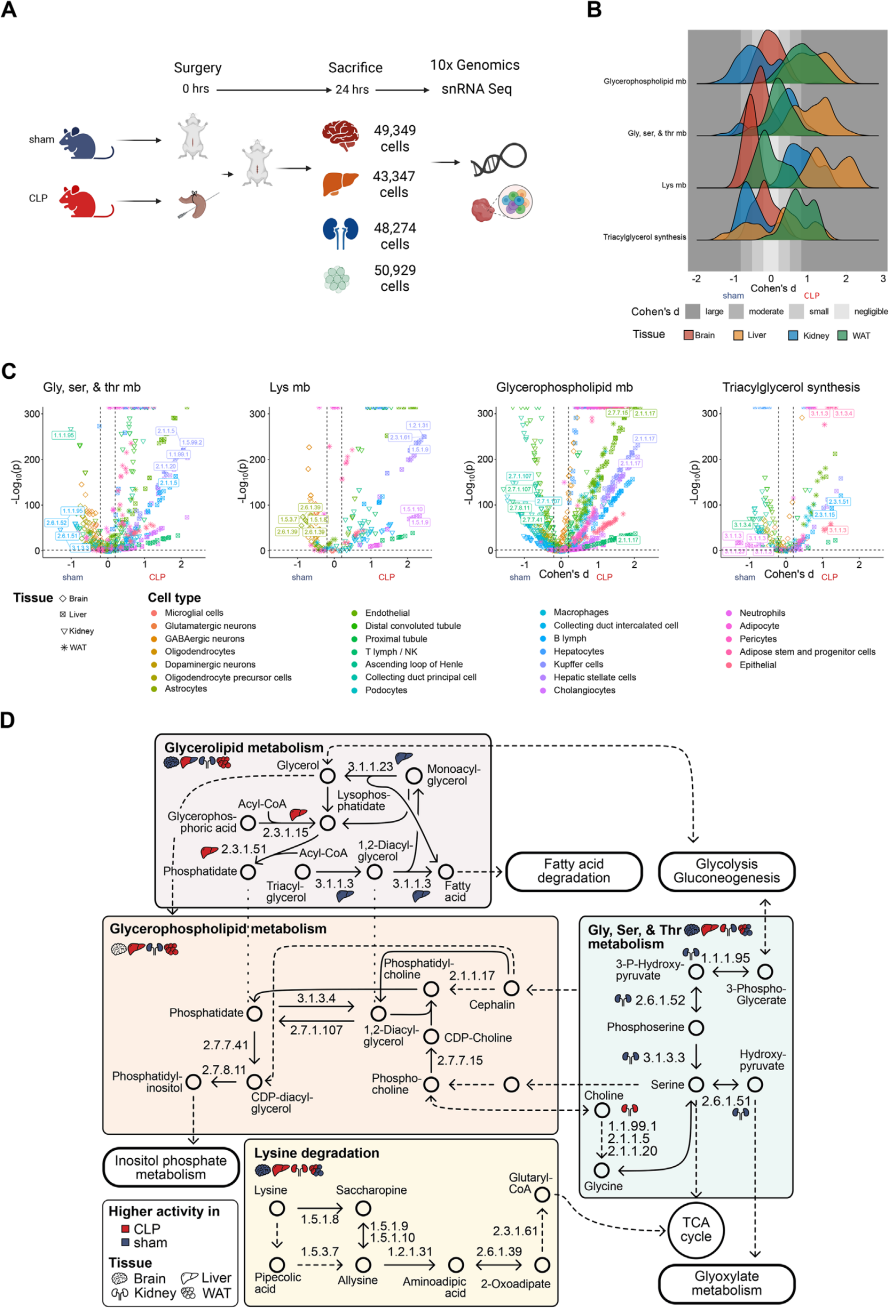
Single cell mouse RNA sequencing driven Compass analysis to identify tissue‐ and cell type‐specific metabolic activity. A) Single‐nucleus RNA sequencing (snRNA‐Seq) design. Triplicates of sham and CLP mice were sacrificed 24 h after operation. Tissue samples of brain, liver, kidney and WAT were collected and yielded between 43347 and 50929 cells in total per tissue. Created with Biorender.com. B) Cohen's d effect size distribution for four energy relevant pathways. Effect sizes by Cohen's d comparing CLP versus sham per pathway associated reaction as defined in the human genome‐scale metabolic model RECON2 across all cell types associated to a given tissue. Different grey background areas reflect different effect sizes. Only reactions with significant changes for CLP versus sham (two‐sided Wilcoxon test, FDR adjusted p ≤ 0.05) were considered. C) Volcano plot for reactions associated to the pathways shown in (B). Y axis shows significance following two‐sided Wilcoxon test of the change CLP versus sham per reaction (FDR adjusted p ≤ 0.05). The top 5 reactions with highest effect change and EC classification number per pathway are indicated. D) Contextualized metabolic network information for highlighted reactions shown in (C). Metabolic network was adapted from the KEGG pathways (https://www.genome.jp/kegg/) hsa00310 (Lysine degradation), hsa00561 (Glycerolipid metabolism), hsa00564 (Glycerophospholipid metabolism) and hsa00260 (Glycine, serine and threonine metabolism). Abbreviations: Gly, ser, ala & thr mb: Glycine, serine and threonine metabolism; Lys mb: Lysine metabolism, Glycerophospholipid mb: Glycerophospholipid metabolism. X‐axis labels sham and CLP reflect direction of change of calculated effect sizes. EC numbers: 1.1.1.95: Phosphoglycerate dehydrogenase; 1.1.99.1: Choline dehydrogenase; 1.2.1.31: Aminoadipate semialdehyde dehydrogenase; 1.5.1.10: Saccharopine dehydrogenase; 1.5.1.8: Saccharopine dehydrogenase (NADP+, L‐lysine‐forming); 1.5.1.9: Saccharopine dehydrogenase (NAD+, L‐glutamate‐forming); 1.5.3.7: L‐pipecolate oxidase; 1.5.99.2: Dimethylglycine dehydrogenase; 2.1.1.17: Phosphatidylethanolamine N‐methyltransferase; 2.1.1.20: Glycine N‐methyltransferase; 2.1.1.5: Betaine‐homocysteine S‐methyltransferase; 2.3.1.15: Glycerol‐3‐phosphate acyltransferase; 2.3.1.51: Lysophosphatidic acid‐acyltransferase; 2.3.1.61: Dihydrolipoamide succinyltransferase; 2.6.1.39: 2‐aminoadipate transaminase; 2.6.1.51: L‐Serine:pyruvate aminotransferase; 2.6.1.52: Phosphoserine transaminase; 2.7.1.107: Diacylglycerol phosphate kinase; 2.7.7.15: Choline phosphate phosphatase; 2.7.7.41: Phosphatidate cytidylyltransferase; 2.7.8.11: Phosphatidylinositol synthase; 3.1.1.23: Acylglycerol lipase; 3.1.1.3: Triacylglycerol lipase; 3.1.3.3: Phosphoserine phosphatase; 3.1.3.4: Phosphatidic acid phosphatase.

We next performed snRNA‐seq on septic (CLP) and control (sham) mice to characterize metabolic responses of different cell types in liver, kidney, WAT, and brain, resulting in 49349; 43347; 48274, and 50929 cells, respectively (**Figure** **4**A). Insufficient high‐quality snRNA data prevented downstream analysis with spleen and heart tissue. Differential gene expression analysis was performed to identify marker genes for cell type annotation and definition, this determined 8, 10, 8, and 6 cell types in liver, kidney, brain, and WAT, respectively (Figure S5, Supporting Information, 26 different cell types and 32 cell type‐tissue combinations in total).

We explored the metabolic activity of the identified key presymptomatic sepsis signatures at the cell type level using the Compass algorithm (see Experimental Section for details). To specifically analyze the metabolic context in sepsis of our observed relevance of serine and aminoadipic acid linked with lipid metabolism, we focused on the subsystems “Glycine, serine and threonine metabolism”, “Lysine metabolism”, “Glycerophospholipid metabolism” and “Triacylglycerol synthesis” as defined in the human genome‐scale metabolic reconstruction RECON2 (Figure 4B, Experimental Section). In total, 3566 comparisons of Compass scores were computed for CLP versus sham over all 32 different tissue‐cell type combinations. The effect size of each Compass score change per tissue and cell type was assessed by Cohen's d analysis, of which 3126 were significant (FDR ≤ 0.05) and subject to the subsequent analyses.

A distribution analysis revealed elevated metabolic activity probabilities in liver CLP samples in the investigated four subsystems with the exception of triacylglycerol synthesis, which showed widespread density distribution and thus cell‐type specific effects (Figure 4B). The highest metabolic induction in septic CLP mice was in lysine metabolism in liver, where aminoadipic acid acted as the hub in inter‐tissue communication (Figure 3D). WAT cells showed elevated metabolic activity probabilities in glycerophospholipid metabolism and triacylglycerol synthesis corroborating their role in lipid metabolism. Kidney cells, on the other hand, were characterized in sepsis by decreased predicted metabolic activity in glycerophospholipid metabolism and triacylglycerol synthesis, and increased metabolic activity in lysine metabolism (Figure 4B). Brain cells were generally less affected and showed a moderate decrease in lysine metabolism in septic CLP mice (Figure 4B).

To identify the most pronounced cell‐type specific drivers of changes at the metabolic level, the top five most changed reactions for CLP versus sham per pathway for which an enzymatic classification by Enzyme Commission (EC) number was available were further investigated (Figure 4C; Table S5, Supporting Information). In all liver cells during sepsis the reactions associated with degradation of lysine to aminoadipic acid, with glutamate as the side‐product, were profoundly activated (Figure 4C). Among all liver cell types, the enzymes alpha‐aminoadipic semialdehyde synthase (AASS, EC 1.5.1.8, 1.5.1.9), saccharopine dehydrogenase (SDH, EC 1.5.1.10), and aminoadipate semialdehyde dehydrogenase (ASDH, EC 1.2.1.31) showed the highest predicted activity increase in liver‐associated cholangiocytes (Cohen's d ≥ 2.09), Kupffer (Cohen's d ≥ 2.23), and stellate cells (Cohen's d ≥ 2.02) (Figure 4C), suggesting that these liver cell types are the main contributors to the metabolic hub function of liver aminoadipic acid (Figure 3D; Table S5, Supporting Information).

In WAT cells, the metabolic activity shift was generally diverse within the investigated pathways (Figure 4C). For instance, during sepsis WAT lysine degradation was reduced in WAT pericytes (Cohen's d in [−0.64, −0.24]), but increased in WAT epithelial cells (Cohen's d in [0.28, 0.67], Table S5, Supporting Information), hinting toward at a protective role of glutamine^[^ ^38^ ^]^ to selected WAT cells. In support of our prior observation of serine and its involvement in WAT metabolism, Compass predicted significantly increased metabolic activity of serine‐associated enzymatic reactions (among others, ECs 2.1.1.5, 1.1.99.1, and 3.1.3.3) in selected WAT cell types (Figure 4C), including epithelial cells (Cohen's d up to in [0.62, 0.85]), adipose stem and progenitor cells (Cohen's d in [0.36, 0.63]), and in part also in adipocytes (Cohen's d = 0.27 for EC 2.1.1.5 and 0.33 for 1.1.99.1, respectively; Table S5, Supporting Information). Likewise, WAT epithelial, adipose stem and progenitor cells also exhibit increased activity in two further key metabolic reactions in serine metabolism, phosphoglycerate dehydrogenase (PGDH, EC 1.1.1.95, Cohen's d ≥0.4) and serine‐pyruvate aminotransferase (EC 2.6.1.51, Cohen's d ≥0.4) in sepsis (Table S5, Supporting Information). This supports an important role of WAT epithelial and adipose stem and progenitor cells with respect to serine‐driven lipid metabolism during sepsis. In contrast, WAT pericytes showed decreased activity of these enzymes in sepsis, suggesting a complex regulation of the associated serine hydroxymethyl transferase (SHMT) cycle in WAT. In addition, triacylglycerol Lipase (ATGL, EC 3.1.1.3), which converts TGs and DGs to fatty acids and glycerol, showed increased activity of different degree over all WAT cells (Figure 4C).

By exploring potential metabolic cross‐talk across the investigated pathways by Spearman correlations of the Compass scores, the T lymphocytes among all liver cells showed a pronounced correlation decrease in sepsis compared to other liver cell types (Figure S6, Supporting Information). While kidney cells showed an overall increase in metabolic crosstalk (especially in endothelial cells and macrophages), brain cells (most pronounced in GABAergic and dopaminergic neuronal cells), and especially all WAT cell types, showed a loss of correlation across all investigated pathways in sepsis. This hints at crosstalk challenges for the liver adaptive immune response and WAT in lipid, lysine and serine metabolism (Figure S6, Supporting Information).

We next contextualized our identified most changing metabolic reactions with pathway information from the Kyoto Encyclopedia of Genes and Genomes (KEGG, Figure 4D). Next to the interconnected role of lysine degradation and aminoadipic acid toward energy replenishment via the TCA cycle, serine and cholines are central within this network. This analysis highlights their role, and network perturbation during sepsis, and in glycero(phospho)lipid metabolism in the metabolic response to sepsis.

In summary, snRNA‐data‐driven Compass simulations suggest significant changes in a number of key metabolic reactions, particularly within liver Kupffer cells and WAT epithelial cells. Notably, changes in the SHMT cycle and lysine degradation suggest elevated roles for converting involved metabolites, including serine or aminoadipic acid, to countereffect energy shortages and, potentially, mitochondrial dysfunction in sepsis.

### 2.6 Tissue‐Independent as well as ‐Specific Adaptations of Energy Metabolism

**Figure 5:**
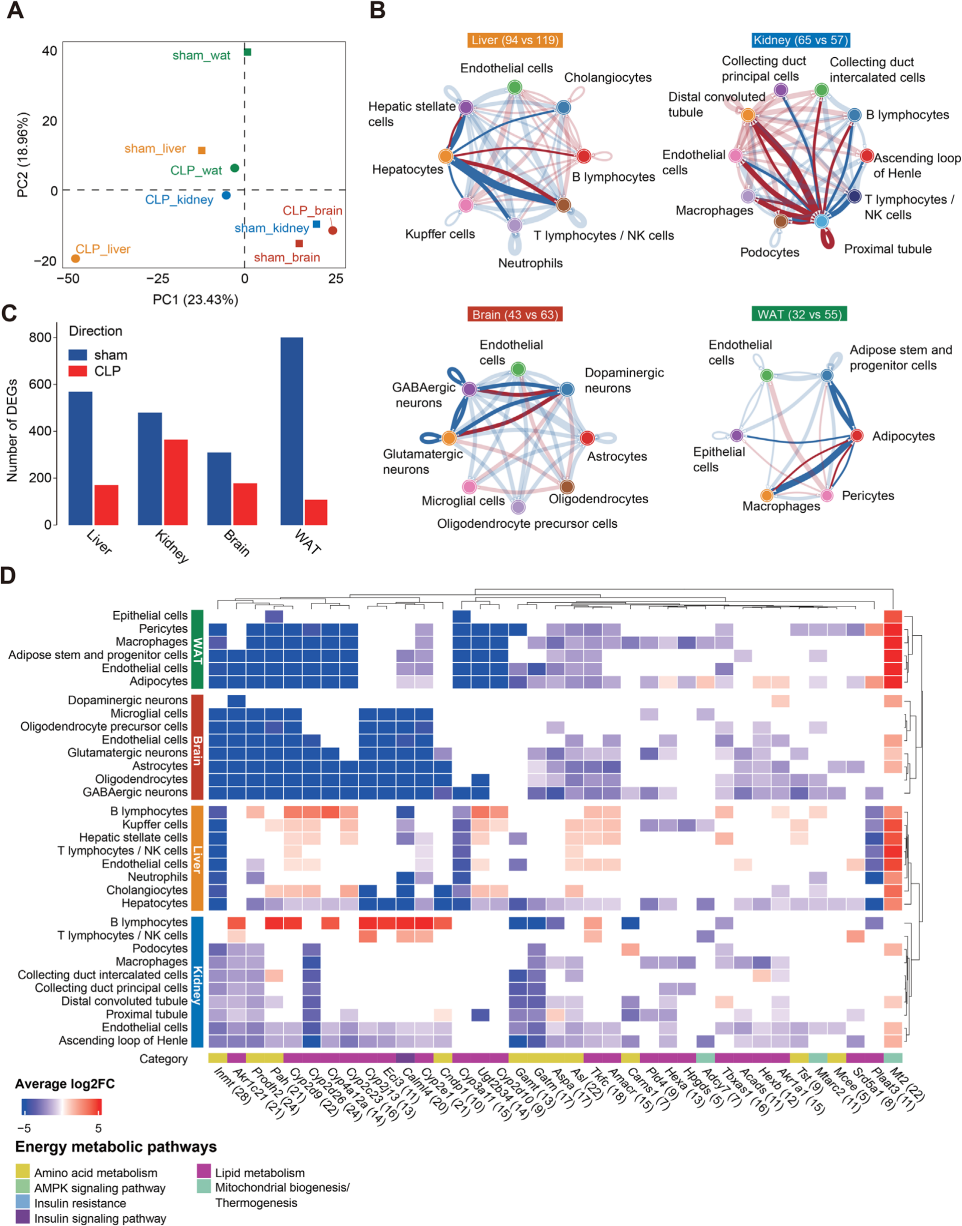
Mouse tissue‐ and cell type‐Specific gene regulation of energy metabolic pathways in CLP‐induced sepsis. A) Principal component analysis (PCA) based on the average gene expression profiles of energy metabolic genes in the four tissues. Axes reflect the first two principal components (PC1, PC2) and their explained variance. B) Cell‐cell communication of genes belonging to the 34 energy‐related pathways based on the KEGG database in different cell types of the four tissues. Numbers in the tissue label indicate the number of energy‐related gene‐gene interactions for CLP versus sham. Linkages connecting the key metabolic cell types of hepatocytes, proximal tubule, neurons, and adipocytes are highlighted by saturated color. Red and blue linkage colors indicate more interaction pairs in CLP or sham, respectively. Linkage width is associated with the number of observed interactions between any two connected cell types. C) Number of differentially expressed genes (DEGs) between CLP and sham in the four tissues. Red and blue represent up‐ and down‐regulated genes in CLP and sham, respectively. D) Average log_2_(fold‐change) profile of the 37 DEGs with consistent variation direction across four tissues at the cell type level between CLP and sham. Red and blue colors indicate upregulated genes in CLP and sham, respectively.

The evidence of metabolic shifts in presymptomatic patients and the predicted rewired activity of specific metabolic sub‐systems in the CLP mouse model suggested that energy deficiency in early sepsis is different across tissues and cell types. To explore the potential regulatory component, we investigated energy‐metabolism‐related gene alterations across different tissues and cell types based on the mice snRNA‐Seq data. A total of 1473 genes associated with 34 bioenergetic‐related pathways as defined by the KEGG database (Table S6, Supporting Information) were identified (liver: 1354; kidney: 1357; brain: 1364; WAT: 1384). The overall variation in energy metabolism‐related genes, based on tissue‐specific average gene expression, clearly separated between CLP and sham‐treated animals for all tissues with the least variation in brain tissue (**Figure** **5**A).

We further constructed intercellular communication networks within each tissue using CellChat^[^ ^39^ ^]^ to investigate energy‐metabolism relevant cell‐cell signaling variations in sepsis (see Experimental Section for details). In general, we found reduced cell‐cell communications in CLP compared to sham mice in liver (94 versus 119), brain (43 versus 63), and WAT (32 versus 55) tissues, while kidney displayed the opposite (65 versus 57) (Figure 5B). At the cell type level, there were decreased numbers of interactions with CLP in liver hepatocytes, brain neurons, and WAT adipocytes (Figure 5B). These findings, combined with the consistent decrease in metabolic cross‐talk of serine‐ and aminoadipic acid‐related reactions in the corresponding cell types (Figure S6, Supporting Information), highlight the potentially critical contributions of serine and aminoadipic acid to the overall decay of energy‐related cell‐cell communications across these three cell types.

To further investigate the responses in mice tissues during sepsis, we performed differential expression analysis and identified 741, 840, 511, and 905 energy‐related differentially expressed genes (DEGs) in liver, kidney, brain, and WAT, respectively (Figure 5C). We observed less up‐regulated DEGs in CLP compared to sham irrespective of the assessed tissue. We then analyzed expression patterns by hierarchical clustering of energy‐related pathways at the cell type level. We found distinct expression patterns for liver together with kidney, as well as brain together with WAT cells (Figure S7A, Supporting Information). These differences corresponded to the tissue‐specific pattern of mitochondrial biogenesis and thermogenesis related genes, with up‐regulated expression in all WAT cell types but down‐regulated expression in most kidney cell types, except for B lymphocytes (Figure S7A, Supporting Information). In addition, different energy‐related pathway expression patterns were found across cell types in each examined tissue. While liver neutrophils and hepatocytes showed down‐regulation of energy‐metabolism‐related pathways, potentially accounting for the overall reduction of energy metabolism in liver tissue, most other liver cell types showed an effect in the opposite direction (Figure S7A, Supporting Information). Interestingly, serine metabolism‐associated *Shmt1* expression was up‐regulated in most liver cell types, including immune cells such as B lymphocytes and Kupffer cells (Figure S7B, Supporting Information), and significantly down‐regulated in hepatocytes. Genes associated with lysine degradation were up‐regulated, especially in cholangiocytes, Kupffer, and stellate cells in liver, and in all kidney cell types, but down‐regulated in all brain cell types, and in WAT endothelial cells and macrophages (Figure S7A, Supporting Information), adding a consistent regulatory component to our metabolism‐focused Compass results (Table S5, Supporting Information).

To explore whether there are also tissue‐independent transcriptional changes of genes contributing to energy regulation and metabolism in response to sepsis, we investigated DEGs for consistent direction of change. Thirty‐seven energy metabolism‐associated DEGs were identified with the same transcriptional changes in CLP compared to sham mice across all four tissues (Figure S7C, Supporting Information). Except for *Mt2*, a mitochondrial biogenesis/thermogenesis‐related gene with antioxidant function,^[^ ^40^ ^]^ all 22 DEGs, which are associated with lipid metabolism, were down‐regulated in sepsis. For instance, the glycerolipid metabolic gene *Akr1a1*, a cancer biomarker associated with mitochondrial metabolic activity,^[^ ^41^ ^]^ was down‐regulated across all tissues (Figure S7C, Supporting Information), again pointing to overall mitochondrial adaptation and dysfunction during sepsis. Apart from up‐regulated *Mt2*, two further mitochondrial biogenesis/thermogenesis‐related DEGs, *Adcy7* and *Mtarc2*, were consistently down‐regulated at the tissue level in septic mice (Figure S7C, Supporting Information), reflecting the dominating effect direction we see at the cell type level per tissue (Figure 5D). Interestingly, the serine‐related DEGs *Gamt* and *Gatm* also exhibited consistent down‐regulation in septic mice across cell types in all tissues.

Overall, next to tissue‐independent we also observed tissue‐specific transcriptional adaptation of energy metabolic genes across all four tissue sites and their cell types during sepsis in mice. Furthermore, we identified tissue‐independent differential regulation of specific mitochondrial biogenesis and thermogenesis genes, including *Mt2*, *Adcy7*, and *Mtarc2*. By using tissue‐specific mouse data, we could add another facet to the mounting evidence for systemic alterations in energy metabolic responses during sepsis.

### 2.7 Identification of Hub Genes Linking Mitochondrial Biogenesis with Sepsis‐Induced Lipid Metabolism Dysregulation

**Figure 6:**
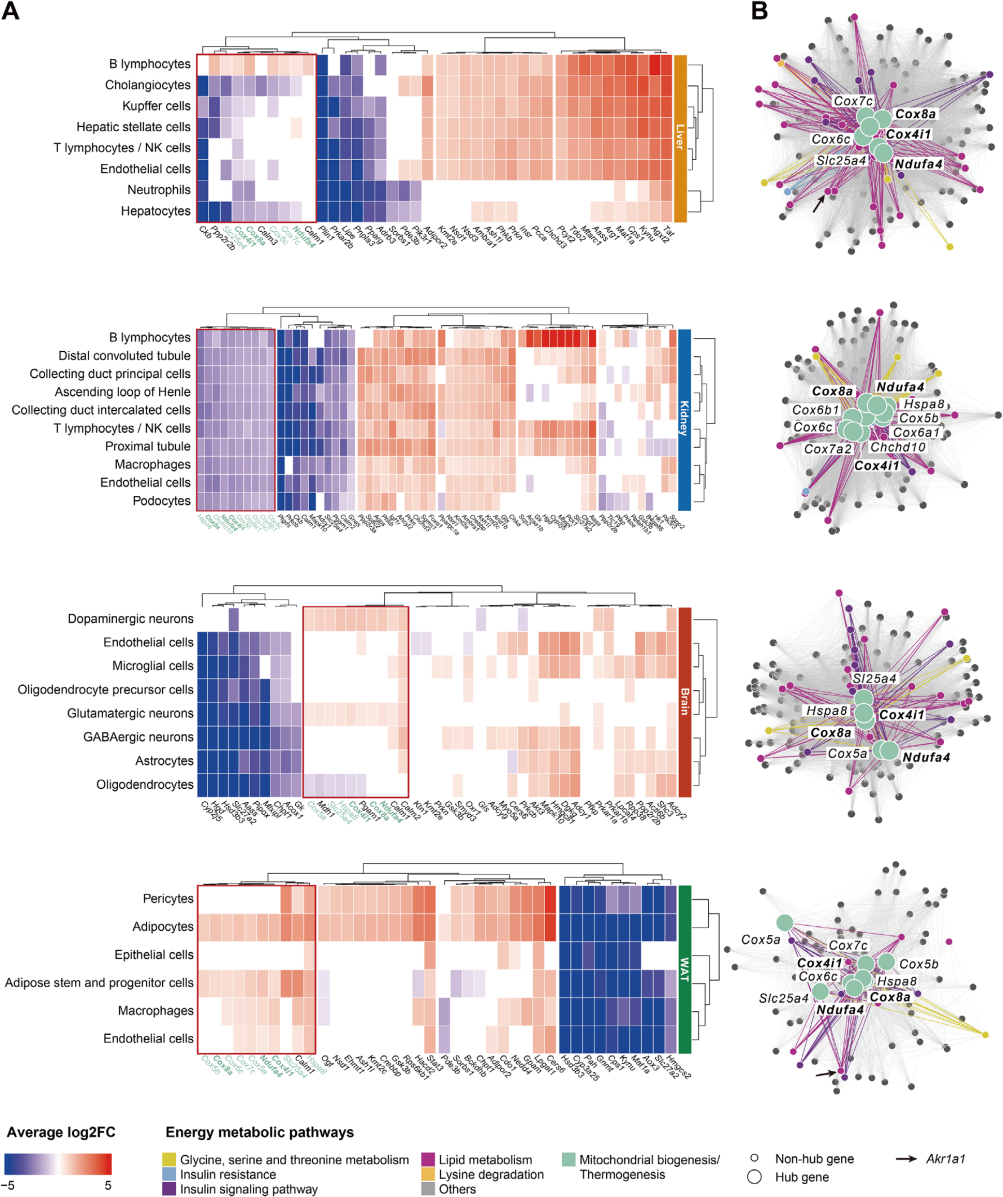
Gene co‐expression networks associated with energy metabolism and hub genes in mouse CLP‐induced sepsis. A) Average log_2_(fold‐change) profile of the hub genes in all modules per tissue for CLP versus sham. Red and blue colors indicate up‐ and down‐regulated genes in CLP, respectively. The structurally common module across all four tissues is indicated by a red border. Hub genes belonging to the mitochondrial biogenesis/thermogenesis pathway are highlighted in green, within which the three common genes across tissues are in bold font. B) Co‐expression networks based on energy metabolism associated genes in the common module per tissue. Each node represents one gene, and each edge refers to the co‐expression relationship between two connected nodes. Node color reflects pathway association. Nodes of hub genes belonging to mitochondrial biogenesis/thermogenesis are enlarged and labeled with gene symbols. Colored linkages indicate the pathway origin of genes linked with mitochondrial biogenesis/thermogenesis hub genes. The three hub genes *Cox4i1*, *Cox8a* and *Ndufa4* present in each tissue‐specific network are indicated in bold font. *Akr1a1* was labeled with a black arrow in the liver and WAT.

To provide a more comprehensive understanding of the transcriptional regulation of energy‐related metabolic genes, we constructed and analyzed gene co‐expression networks for each tissue. We identified 4, 6, 5, and 4 gene co‐expression modules (by weighted gene co‐expression network (WGCNA), see Experimental Section for details) in liver, kidney, brain, and WAT, respectively (**Figure** **6**A). Of note, we observed one module, which we refer to as the common module, in every tissue where most of the hub genes (highly connected genes within a module) were associated with mitochondrial biogenesis/thermogenesis function (Figure 6A, cluster with red border). In addition, most non‐hub genes in the common module also associated with mitochondrial biogenesis/thermogenesis function, displaying a pronounced co‐expressed gene interaction across all tissues toward mitochondria‐related energy metabolism in the septic mice (Figure S7D, Supporting Information). Strikingly, three of the mitochondrial genes, *Cox4i1*, *Cox8a*, and *Ndufa4*, were hub genes in the common module in all four tissues (Figure 6A). *Cox4i1* and *Cox8a* encode for subunits of cytochrome c oxidase while *Ndufa4* encodes for a subunit of nicotinamide adenine dinucleotide hydrogen (NADH) dehydrogenase. These enzymes are essential parts of the mitochondrial electron transport chain (ETC), contribute to adenosine triphosphate (ATP) generation, and have been linked to thermogenesis as alternative output of mitochondrial respiration.^[^ ^42^ ^]^

While the structure of the common module appeared independent of tissue type, a tissue‐specific co‐expression pattern in liver and kidney was observed. Here, all hub genes in the common modules were down‐regulated in sepsis across all cell types except B lymphocytes within liver (Figure 6A). In contrast, with the exception of brain oligodendrocytes, all brain and WAT cell types up‐regulated the hub genes of the common module upon early sepsis. We also identified tissue‐independent hub gene co‐expression with multiple genes associated with serine metabolism, lysine degradation, and lipid metabolism (Figure 6B). Among these, lipid metabolism associated genes were the most connected to the mitochondrial biogenesis/thermogenesis hub genes. *Akr1a1*, identified as one of the 36 tissue‐independent down‐regulated genes (Figure S7C, Supporting Information), was co‐expressed with all 15 mitochondrial biogenesis/thermogenesis hub genes in liver and WAT (Figure 6B, black arrow). Genes involved in glycerolipid and glycerophosphlipid metabolism (Table S7, Supporting Information) were also co‐expressed with hub genes from the common modules, including *Agpat5* (lysophosphatidic acid‐acyltransferase, EC 2.3.1.51) and *Cdipt* (phosphatidyltransferase, EC 2.7.8.11). These insights corroborate their role in energy metabolic adaptations we predicted with Compass particularly in liver and WAT, but not in kidney cells in septic mice (Figure 4C and 4D). Since both processes are vital for lysophosphatidic acid (LPA) to PA conversion, mitochondrial membrane integrity, and PI synthesis,^[^ ^43^ ^]^ our findings suggest that kidney cells require modulation of PIs during sepsis to ameliorate tissue damage protection. This is in support of the kidney tissue protective role of PI phosphorylation in a murine sepsis model.^[^ ^44^ ^]^

Taken together, our co‐expression insights underline the potential co‐regulation of mitochondrial ETC‐mediated ATP generation with other energy‐related metabolic pathways, particularly including lipids associated with membrane integrity.

### 2.8 Labeled Serine Administration Reveals Drop in Hepatic PCs and Increase in Purine Metabolism

**Figure 7:**
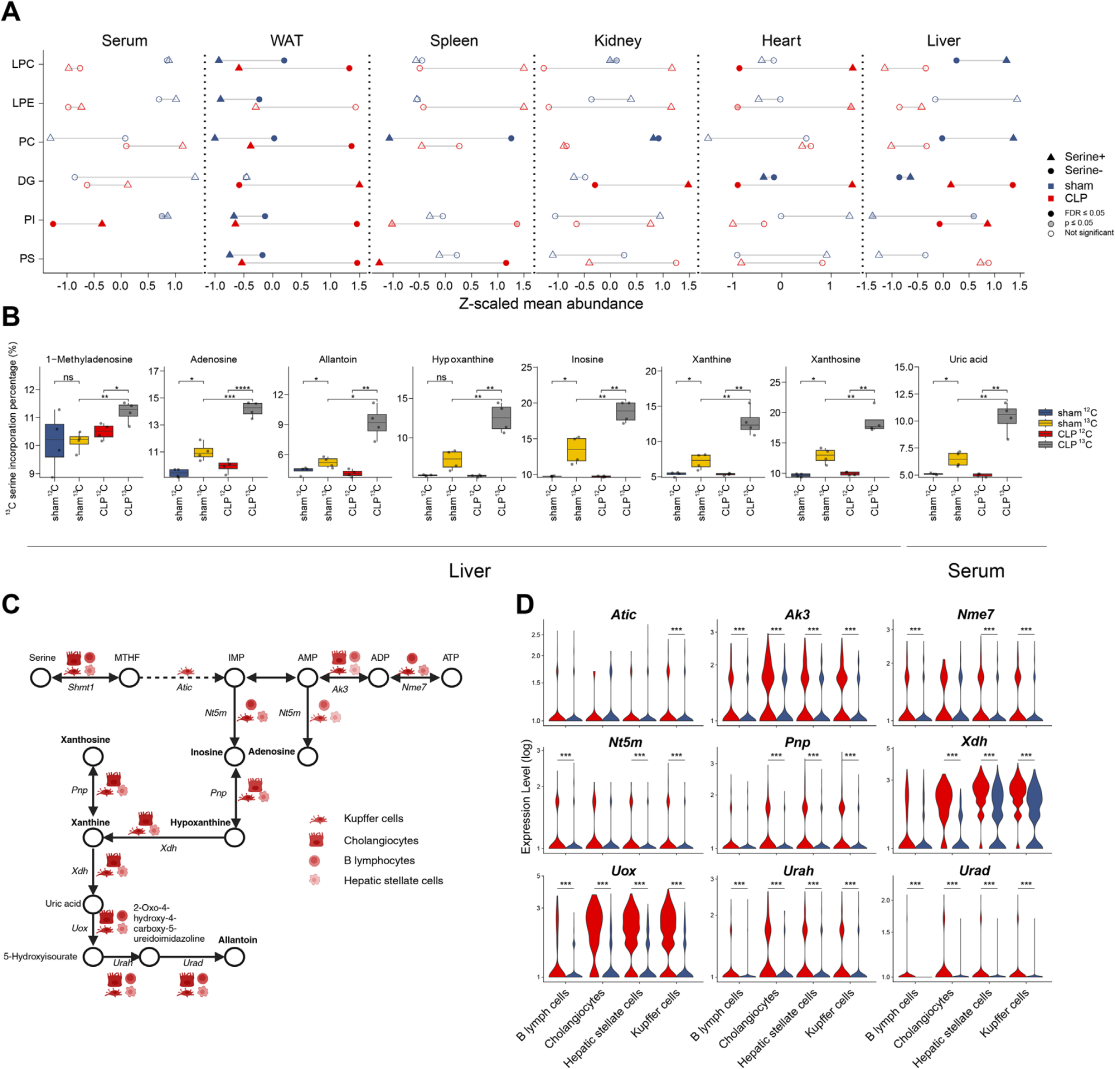
Serine administration and serine labeling experiments reveal drop in hepatic PC and elevated serine integration into purines in early sepsis. A) Differential lipid abundance in Serine+ versus Vehicle (Serine‐) treated mice in both CLP and sham groups. Mean abundance is z‐scaled. B) Metabolites with significantly incorporated ^13^C‐labeled serine‐derived carbon in septic mice. Only metabolites with significant higher incorporation percentage in ^13^C CLP than ^13^C sham (one‐sided Student's t test, *p* ≤ 0.05, FDR adjusted p ≤ 0.25) and higher incorporation percentage in ^13^C CLP than ^12^C CLP (one‐sided Student's t test, FDR adjusted p ≤ 0.05) were considered. C) Schematic of purine metabolism in mice liver in context of tissue specific gene expression profile based on snRNA data. Metabolic network was adapted from the KEGG pathways (https://www.genome.jp/kegg/) mmu00260 (Glycine, serine and threonine metabolism) and mmu00230 (Purine metabolism). All cell types shown next to genes indicated up‐regulated gene expression in these cells in liver of septic compared to sham mice. Created with Biorender.com. D) Violin plot of nine genes in purine metabolism shown in (C). ^*^ *p* ≤ 0.05; ^**^ *p* ≤ 0.01; ^***^ *p* ≤ 0.001. LPC: lysophosphatidylcholine; LPE: lysophosphatidylethanolamine; PC: phosphatidylcholine; DG: diglyceride; PI: phosphatidylinositol; PS: phosphatidylserine.

Until this point, we accumulated evidence for serine involvement in the energy adaptive processes in sepsis. This included the reduction in serine levels and its inverse correlation with infection severity in our human presymptomatic *Sepsis* group, as well as altered serine‐related metabolic activity and gene expression at the tissue and cell level in septic mice. Consequently, we further investigated the location and impact of serine‐dependent alterations of metabolic mechanisms in sepsis. First, we investigated the impact of serine on sepsis at the tissue level by administering unlabeled serine to sepsis (CLP) and control (sham) mice (**Figure** **7**A, see Experimental Section for details). Most pronounced, we found altered lipid levels upon serine addition. Heart tissues showed significant increases in LPCs and DGs only in septic mice (FDR ≤ 0.05, Cohen's d in [1.22, 1.39]), while liver LPCs, PCs, and DGs increased only in control mice (FDR ≤ 0.05, Cohen's d in [0.43, 0.97]) (Figure 7A). All further tissues and serum showed less pronounced differences between control and sepsis mice (Figure 7A). While spleen and kidney PCs were significantly reduced in control mice (FDR ≤ 0.05, Cohen's d in [−0.37, −0.46]) upon serine addition, septic mice only showed a trend, whereas serum and WAT showed sepsis‐independent alteration of serine induced lipid changes (e.g., increased PIs in both sepsis and control serum, or reduced LPCs, PCs, PIs, and phosphatidylserines (PSs) in WAT, Table S8, Supporting Information).

To investigate more precisely in which metabolic components serine is integrated, we orally administered ^13^C‐labeled serine to both control and sepsis mice (see Experimental Section for details). We first quantified the enrichment of downstream lipids in serum and five tissues (Table S9, Supporting Information). ^13^C‐labeled serine‐derived carbon was incorporated significantly into liver LPCs (Fisher's exact test, *p* ≤ 0.05) and PCs (Fisher's exact test, *p* ≤ 0.05, FDR ≤ 0.1) in control but not in sepsis mice (Table S9, Supporting Information), demonstrating perturbed serine associated PC biosynthesis in liver during sepsis.

Interestingly, when we investigated further metabolites involved in the direct incorporation of serine, we found that ^13^C‐labeled serine‐derived carbon was significantly incorporated into purine metabolites in the liver of septic mice (Figure 7B; Table S2, Supporting Information). Across serum and five tissues, eight metabolites showed significantly higher incorporation of ^13^C‐labeled serine‐derived carbon in sepsis compared to control mice (Student's t‐test: *p* ≤ 0.05, FDR ≤ 0.25, Cohen's d ≥1.7) (Figure 7B). All of these metabolites were associated with purine metabolism in the liver. In serum, only uric acid showed significantly increased incorporation of ^13^C‐labeled serine‐derived carbon in septic mice (Figure 7B).

By cross‐checking these insights with our snRNA data, we also found up‐regulation of genes involved in purine metabolism in liver, particularly those linked to converting metabolites incorporating ^13^C‐labeled serine‐derived carbon in Kupffer cells, cholangiocytes, B lymphocytes, and hepatic stellate cells (Figure 7C). Downstream of *Shmt1*, particularly *Ak3*, *Uox*, *Urah*, and *Urad* were consistently up‐regulated across all four liver cell types (Figure 7D).

We conclude that next to a serine‐specific effect on the abundance of lipids, purine biosynthesis, and salvage processes are differentially regulated in sepsis, potentially as a response to the energetic shortages given mitochondrial malfunction.

## 3 Discussion

Metabolic adaptation is crucial during sepsis progression to provide substrates for biosynthetic pathways, and to regulate signaling and organ function with the aim of coping with the allostatic load and eventually re‐establishing cellular homeostasis.^[^ ^11^, ^45^ ^]^ Several studies explored the potential of metabolic markers for sepsis,^[^ ^16^, ^17^, ^46^, ^47^, ^48^ ^]^ and presented machine learning models that can predict sepsis mortality with high accuracy. However, these studies primarily utilize blood samples from patients with established sepsis, where adaptive and maladaptive responses likely coexist and are complicated further by factors associated with organ dysfunction and confined body regions for immune responses.^[^ ^22^, ^49^, ^50^ ^]^ Here, we utilized a longitudinal cohort of serum samples taken from presymptomatic patients undergoing elective surgery to capture the earliest metabolic responses to sepsis (Figure 1). We identified serine, aminoadipic acid, and specific lipid classes (LPCs, LPEs, and PCs) as the most altered metabolic processes (Figure 2).

Using a CLP sepsis mouse model, we identified a tissue‐ and cell type‐specific likelihood of metabolic activity in the serine hydroxymethyltransferase (SHMT) cycle connected to choline and lipid metabolism based on snRNA data and metabolic model analysis (Figures 4 and 5). While the SHMT cycle is essential for providing energy in macrophage polarization,^[^ ^51^ ^]^ cholines have been shown to attenuate sepsis‐associated acute kidney injury.^[^ ^52^, ^53^ ^]^ Serine is also an important regulator of mitochondrial biogenesis and functionality,^[^ ^54^, ^55^ ^]^ in part acting via serine‐derived lipids (ceramides),^[^ ^56^ ^]^ and has been associated with the maintenance of central carbon metabolism and nucleotide metabolism.^[^ ^57^ ^]^ Alongside, we observed increased concentrations of lysine degradation‐associated aminoadipic acid during sepsis, especially in liver cells, which potentially affects lipolysis,^[^ ^58^ ^]^ and mitochondrial biogenesis and function.^[^ ^59^, ^60^ ^]^ In our data, liver aminoadipic acid is central to inter‐tissue communication (Figure 3), supporting its critical role in the metabolic response to infection‐induced multi‐organ dysfunction in sepsis.^[^ ^61^ ^]^ By using metabolic modeling, we further identified liver Kupffer cells and WAT epithelial cells as key drivers of the serine and aminoadipic acid pathways, respectively. Both, serine and aminoadipic acid, are hitherto unrecognized early tissue‐ and cell‐type specific modulators of lipid and mitochondrial metabolic pathways in sepsis and, more importantly, can be linked to metabolic alterations in serum of presymptomatic patients.

Tissue‐ and cell‐type dependent as well as independent regulatory and metabolic shifts suggest effects on mitochondrial biogenesis, associated with altered lipid metabolism. In this context, we identified common co‐expression modules with mitochondrial‐ and thermogenesis‐related genes serving as hubs and linking genes involved again in serine metabolism, lysine degradation, and lipid metabolism across the four investigated tissues (Figure 6). Specifically, we identified *Cox4i1*, *Cox8a*, and *Ndufa4* as three hub genes in the common modules, suggesting an important role for these three genes and their protein products within the electron transport chain, the mitochondrial ATP synthesis, and thermogenesis.^[^ ^42^ ^]^ Of note, all three genes have already been confirmed as drug targets according by the DrugBank database,^[^ ^62^ ^]^ and appear to be relevant also to sepsis according to our data.

Aligned with the elevated SHMT cycle activity predicted by metabolic modeling and the increased transcription of *Shmt1*, our serine intervention model demonstrated that serine shifts toward purine metabolism in the liver of septic mice. This shift is likely facilitated by the SHMT pathway and associated with malfunctioning mitochondrial ETC‐driven energy generation.^[^ ^63^ ^]^ Specifically, the ETC proteins COX4i1 and NDUFA4, whose genes are central in our identified common module with differential tissue‐dependent expression upon sepsis, have been linked to a heme‐dependent switch of mitochondrial ATP synthesis to thermogenesis.^[^ ^42^ ^]^ This link highlights the role, these two proteins potentially play in the metabolic adaptations and tolerance observed in sepsis.^[^ ^8^ ^]^ Additionally, our serine intervention model revealed reduced serine utilization for PC biosynthesis, along with alterations in serine‐associated purine abundances in septic mice (Figure 7). It appears therefore likely that exogenous serine can contribute to maintain purine biosynthesis also in sepsis. This includes an impact of serine levels on the pentose phosphate pathway and TCA cycle with the ultimate goal of increased energy replenishment via mitochondrial biogenesis^[^ ^57^, ^63^, ^64^ ^]^ within hepatic, particularly Kupffer cells to counteract energy deficiency during sepsis.

Our study is not without limitations. Our human cohort enabled us to identify presymptomatic human signatures of sepsis before clinical manifestations. The current uniqueness of our dataset, however, warrants validation of our results in an independent future cohort, ideally including multiple centers with varying ethnicities, and causes of sepsis. This would also be important to better evaluate potentially varying levels of metabolic markers across different patient populations, as well as the influence of other comorbidities, including, e.g., diabetes, or liver dysfunction on metabolite levels. Nevertheless, we cross‐checked the relevance of our identified markers against studies with established sepsis and similar data and sample sites. We find 82% concordance in significance and direction with our human metabolite and lipid signature results compared to cohorts with established sepsis, corroborating the relevance of our identified markers also in presymptomatic sepsis (Table S10, Supporting Information). In particular, our study aligns with the metabolic (67% concordance) and lipidomic signatures (78% concordance considering lipid class level) observed at the enrollment time point in cohorts with community‐acquired sepsis^[^ ^17^ ^]^ and pneumonia‐associated sepsis.^[^ ^65^ ^]^ Our study design, using a presymptomatic patient cohort, already allowed us to reduce potential confounding effects from organ dysfunction or sepsis‐induced catabolism. Furthermore, we minimized the impact of confounding effects by adjusting for age and sex, and by normalizing postoperative data using patient‐resolved preoperative samples. Assessing further physiological processes, such as early changes in renal clearance^[^ ^66^ ^]^ can help rule out potential effects on the metabolic shifts we have described and may help to further stratify patients at risk of developing sepsis.

In conclusion, our analysis provides a comprehensive view on dynamic metabolic adaptations which can be linked to presymptomatic sepsis in human patients. We highlighted key metabolic factors including altered serine and aminoadipic acid metabolism, as well as substantial lipid depletion, which all pinpoint to a bioenergetic deficiency and mitochondrial dysregulation. Targeting our identified tissue‐independent module and its hub genes could potentially improve mitochondrial dysfunction in early sepsis and even offer completely new therapeutic strategies to safeguard patients at risk of developing sepsis.

## 4 Experimental Section

### Study Design

Elective surgery patients were prospectively recruited at seven UK hospitals and one German center between November 2007 and February 2017. Ethical approval was granted through the Southampton and South‐West Hampshire Multicentre Research Ethics Committee (Reference 06/Q1702/152) and registered with ISRCTN (No. 17 375 399).^[^ ^67^ ^]^ The protocol achieved US Federal Wide Assurance Independent Review Board status (IRB00001756). Patients were enrolled if aged between 18 and 80 years, due to undergo elective major abdominal, cardiac, gynae‐urological, thoracic, or vascular surgery, and provided informed consent. All patient information including demographics, type of surgery, and perioperative physiological, biochemical, and microbiological data were compiled into a comprehensive database (Item Tracker Software, Ipswich, UK) by research staff at each center. The patient grouping selection process included a Clinical Adjudication Panel (CAP) as described in detail in Lukaszewski et al.^[^ ^23^ ^]^ Briefly, a CAP of multidisciplinary specialists was established to review and classify postoperative patients based on standardized criteria. Five CAPs were held between July 2013 and March 2017. CAP members independently assessed clinical data for patients suspected of infection, assigning them to categories including *Sepsis*, *Infect*, *SIRS*, and *Control*. Final classifications were reached through group voting and discussion, ensuring more robust and consensus‐based diagnoses.

### Mice

All experimental animal procedures in this study were evaluated through the Independent Ethical Advisory Committee and approved by the Local Government Authority of Thuringia, Thüringer Landesverwaltungsamt, per European and German laws and regulations under the license HKI‐23‐002. For all experiments, male C57BL/6 mice at 26 – 28 weeks of age (obtained from Janvier Labs, France) were housed under specific pathogen‐free (SPF) conditions in the Leibniz Institute for Natural Product Research and Infection Biology (Hans Knöll Institute). All animal experiments follow the European (Directive 2010/63/EU) legislations, concerning housing, husbandry, and animal welfare.

### Cecal Ligation and Puncture

Polymicrobial abdominal sepsis was induced using the CLP model as previously described.^[^ ^8^ ^]^ Mice were anesthetized with a combination of isoflurane and butorphanol (1 mg kg^−1^, s.c.) followed by 30% ligation of the cecum and double puncture with a 23 Gauge needle. A small amount of faeces was extruded and the cecum was placed back into the abdominal cavity. Sham treatment was performed without ligation and puncture. All animals received saline (40 mL/kg, s.c.) directly after the procedure and once Imipenem/Cilastatin (25 mg kg^−1^, s.c.) after 6 h. This model is associated with a 14‐day mortality of 30%, closely mimicking the clinical situation. Animals were orally gavaged with either drinking water (vehicle), 500 mg kg^−1^ 12C‐Serin (Sigma) or 13C‐Serin (CLM‐1574‐H‐0.25 Serine‐L (13C3 99%13C), Euroisotope, Germany) in 10 mL kg^−1^ drinking 6 and 18 h after CLP.^[^ ^68^, ^69^ ^]^ After 24 h, animals were euthanized with an intraperitoneal injection of ketamine/xylazine (500 mg kg^−1^ and 25 mg kg^−1^, respectively, i.p.). The animals were then perfused with ice‐cold Hanks’ Balanced Salt Solution and tissues were harvested, snap‐frozen, and stored at −80° until further analysis.

### Human Serum Sample Preparation

Sample analysis was carried out by MS‐Omics as follows. For semi‐polar metabolite analysis, serum samples (50 µL) were diluted with a mixture of acetonitrile, methanol, formic acid, and stable isotope labeled internal standards (200 µL 1.00:0.99:0.01 v/v/v). The extracts were then passed through a phosphor lipid removal cartridge (Phree, Phenomenex) using centrifugation (1 400 rpm, 4 °C, 10 min). An aliquot (100 µL) was transferred into a high recovery high‐performance liquid chromatography (HPLC) vial and the solvent was removed under a gentle flow of nitrogen. Extracts were reconstituted in a reverse phase mobile phase mixture (100 µL, 10% B in 90% A). For Lipidomics analysis, serum samples (10 µL) were transferred to a Spin‐X filter (0,22 µm, Costar) and diluted in a mixture of isopropanol, SPLASH Lipidomix (Avanti Polar Lipids) and butylated hydroxytoluene (90 µl, 96:4 vol/vol + 10 µg). The extract was then left at room temperature for 10 min before storage at −20 °C overnight. Samples were brought to room temperature and filtered by centrifugation (14 000 rpm, 5 °C, 2 min). An aliquot (25 µL) was transferred into a high recovery HPLC vial and an equal parts mixture of mobile phase eluent A and B (75 µL) was added. For quality control, a mixed pooled sample (QC sample) is created by taking a small aliquot from each sample. This sample is analyzed with regular intervals throughout the sequence.

### Mice Tissue and Serum Sample Preparation

To prepare samples for semi‐polar metabolites extraction, the different tissue samples were weighted and transferred to Eppendorf tubes. Ceramic beads and precooled methanol/water (1:2) were added. All tubes were placed in a pre‐cooled (−20 °C) bead beater and homogenized (4 × 30 sec., 30 Hz) followed by ultrasonication (5 min). After centrifugation (18000 RCF, 5 min, 4 °C), the supernatant was collected. The sample pellet was reextracted as described above. The two extract supernatants were pooled and passed through a phosphor removal cartridge (Phree filter plates). A precise aliquot of the extract was evaporated to dryness under a gentle stream of nitrogen, before reconstitution with 10% Eluent B in Eluent A. All Samples were diluted 5 times in mobile phase eluent A and stable isotope‐labeled standards were added before analysis. Mouse serum (50 µL) was diluted with a mixture of acetonitrile and methanol (200 µL 1:1 v/v). The extract was then passed through a phosphor removal cartridge (Phree, Phenomenex). An aliquot (100 µL) was transferred into a high‐recovery HPLC vial and the solvent was removed under a gentle flow of nitrogen. Extracts were reconstituted in a mobile phase mixture (100 µL, 10%B in 90%A). To ensure high‐quality sample preparation, a quality control sample (QC sample) was prepared by pooling small equal aliquots from each sample, to create a representative average of the entire set. This sample was treated and analyzed at regular intervals throughout the sequence.

In the different tissue samples, the pellet from the semipolar extraction was used to extract lipids as follows. DCM:MeOH (3:1) was added to the pellets, and the samples were homogenized using a pre‐cooled Bead‐beater (4 × 60 sec at 30 Hz), with colling of the blocks between the beading cycles. Subsequently, the samples were ultrasonicated for 5 mins and centrifuged (5 mins, 1800 RCF, 4 °C). The supernatant was collected and dried under nitrogen gas. Samples were then reconstituted with 6x (spleen and WAT) or 3x (heart, liver, and kidney) the sample weight with the reconstitution solvent eluent mix and vortex mixed for 30 sec. The reconstituted samples were filtrated using SpinX filters by centrifugation (5 mins, 1800 RCF, 4 °C). All samples were mixed 1: 50 in eluent mix. For serum samples, an aliquot of the serum samples is mixed with extraction solvent (0.1 M BHT in isopropanol with stable isotope labeled internal standard) in a Spin‐X filter. The filters were vortex‐mixed for 60 sec and left at room temperature for 10 mins before placing in −20 °C Freezer overnight. The following day samples were left at room temperature for 30 mins before centrifuging (1800 g /4 °C/2 min). The filtrate was mixed with eluent mix 1:3.

### Human Semi‐Polar Metabolites and Lipidomics LC‐MS Method

Sample analysis was carried out by MS‐Omics as follows. For semi‐polar metabolites, the analysis was carried out using a Thermo Scientific Vanquish LC coupled to a Q Exactive HF Hybrid Quadrupole‐Orbitrap, Thermo Fisher Scientific. An electrospray ionization interface was used as an ionization source. Analysis was performed in positive and negative ionization mode. The UHPLC was performed using an adapted version of the protocol described by Doneanu et al.^[^ ^70^ ^]^ Metabolomics processing was performed untargeted using Compound Discoverer 3.0 (Thermo Scientific) for peak picking and feature grouping, followed by an in‐house annotation and curation pipeline written in MatLab (2021b, MathWorks). Identification of compounds was performed at four levels; Level 1: identification by retention times (compared against in‐house authentic standards), accurate mass (with an accepted deviation of 3 ppm), and MS/MS spectra, Level 2a: identification by retention times (compared against in‐house authentic standards), accurate mass (with an accepted deviation of 3 ppm). Level 2b: identification by accurate mass (with an accepted deviation of 3 ppm), and MS/MS spectra, Level 3: identification by accurate mass alone (with an accepted deviation of 3 ppm). For lipid analyses, lipidomics high‐performance liquid chromatography (HPLC), coupled with high‐resolution mass spectrometry (HRMS) analysis was performed using a Thermo Scientific Vanquish LC coupled to Thermo Q Exactive HF Hybrid Quadrupole‐Orbitrap mass spectrometer (Thermo Scientific, RRID: SCR_020545). The chromatographic separation of lipids was carried out on a Waters ACQUITY Charged Surface Hybrid (CSH) C18 column (2.1 × 100 mm, 1.7 µm). The column was thermostated at 55 °C. The mobile phases consisted of (A) Acetonitrile/water (60:40) and (B) Isopropanol/acetonitrile (90:10), both with 10 mM ammonium formate and 0.1% formic acid. Lipids were eluted in a two steps gradient by increasing B in A from 40 to 99% over 18 min. The flow rate was kept at 0.4 mL/min. Ionization was performed in positive and negative ionization mode using a heated electrospray ionization interface. The mass spectrometer operated at a resolution of 120000 in a scan range of 200 to 1500 m/z. Iterative data‐dependent MS/MS (dd‐MS^2^) acquisition was achieved on a TopN of 10 with stepped collision energy (NCE) of 20, 40, and 60. Peak areas were extracted using Compound Discoverer 3.0 (Thermo Scientific). Compound annotations were performed against the mzCloud (Thermo Scientific) MSMS library, the Human Metabolome Database 4.0,^[^ ^71^ ^]^ and the MS‐Omics lipid library covering 17 classes (CerP, DG, digalactosyldiacylglycerol (DGDG), Fatty acids, LPC, LPE, monoacylglycerol (MG), PA, PC, phosphatidylethanolamine (PE), phosphatidylglycerol (PG), PI, Plasmenyl‐PC, Plasmenyl‐PE, PS, SM and TG). Identification of compounds was performed at four levels; Level 1: identification by retention times (compared against in‐house authentic standards), accurate mass (with an accepted deviation of 3 ppm), and MS/MS spectra, Level 2a: identification by retention times (compared against in‐house authentic standards and lipid class behavior), accurate mass (with an accepted deviation of 3 ppm). Level 2b: identification by accurate mass (with an accepted deviation of 3 ppm), and MS/MS spectra, Level 3: identification by accurate mass alone (with an accepted deviation of 3 ppm). Lipid annotations were curated manually by comparing retention time behavior over the number of tail chain carbons and double bonds as annotated.

### Mice Semi‐Polar Metabolites and Lipidomics LC‐MS Method

Sample analysis was carried out by MS‐Omics as follows. Semi‐polar metabolite profiling was carried out using a Vanquish LC (Thermo Scientific) coupled to an Orbitrap Exploris 240 MS (Thermo Scientific). The UHPLC was performed using an adapted version of the protocol described by Doneanu et al.^[^ ^70^ ^]^ An electrospray ionization interface was used as ionization source. Analysis was performed in positive and negative ionization mode under polarity switching. Metabolomics processing was performed untargeted using Compound Discoverer 3.3 (Thermo Scientific) and Skyline 22.2^[^ ^72^ ^]^ (MacCoss Lab Software) for peak picking and feature grouping, followed by an in‐house annotation and curation pipeline written in MatLab (2022b, MathWorks). Identification of compounds was performed at four levels; Level 1: identification by retention times (compared against in‐house authentic standards), accurate mass (with an accepted deviation of 3 ppm), and MS/MS spectra, Level 2a: identification by retention times (compared against in‐house authentic standards), accurate mass (with an accepted deviation of 3 ppm). Level 2b: identification by accurate mass (with an accepted deviation of 3 ppm), and MS/MS spectra, Level 3: identification by accurate mass alone (with an accepted deviation of 3 ppm). For lipid analysis, lipidomics high‐performance liquid chromatography (HPLC), coupled with high‐resolution mass spectrometry (HRMS) analysis was performed using a Thermo Scientific Vanquish LC coupled to Thermo Q Exactive HF Hybrid Quadrupole‐Orbitrap mass spectrometer (Thermo Scientific, RRID: SCR_02 0545). The chromatographic separation of lipids was carried out on a Waters ACQUITY Charged Surface Hybrid (CSH) C18 column (2.1 × 100 mm, 1.7 µm). The column was thermostated at 55 °C. The mobile phases consisted of (A) Acetonitrile/water (60:40) and (B) Isopropanol/acetonitrile (90:10), both with 10 mM ammonium formate and 0.1% formic acid. Lipids were eluted in a two‐step gradient by increasing B in A from 40 to 99% over 18 min. The flow rate was kept at 0.4 mL min^−1^. Ionization was performed in positive and negative ionization mode using a heated electrospray ionization interface. The mass spectrometer operated at a resolution of 120 000 in a scan range of 200–1500 m z^−1^. Iterative data‐dependent MS/MS (dd‐MS^2^) acquisition was achieved on a TopN of 10 with stepped collision energy (NCE) of 20, 40, and 60. Peak areas were extracted using Compound Discoverer 3.2 (Thermo Scientific) and Skyline 21 (MacCoss Lab Software). Compound annotations were performed against the mzCloud (Thermo Scientific) MSMS library, the Human Metabolome Database 4.0, and the MS‐Omics lipid library covering 17 classes (CerP, DG, DGDG, Fatty acids, LPC, LPE, MG, PA, PC, PE, PG, PI, Plasmenyl‐PC, Plasmenyl‐PE, PS, SM, and TG). Identification of compounds was performed at four levels; Level 1: identification by retention times (compared against in‐house authentic standards), accurate mass (with an accepted deviation of 3 ppm), and MS/MS spectra, Level 2a: identification by retention times (compared against in‐house authentic standards and lipid class behavior), accurate mass (with an accepted deviation of 3 ppm). Level 2b: identification by accurate mass (with an accepted deviation of 3 ppm), and MS/MS spectra, Level 3: identification by accurate mass alone (with an accepted deviation of 3 ppm). Lipid annotations were curated manually by comparing retention time behavior over the number of tail chain carbons and double bonds as annotated. Stable isotope incorporations (^13^C) were determined against unlabeled reference samples (containing no ^13^C‐substrate). Corresponding compound pairs (labeled versus unlabeled) were determined based on their retention time, accurate mass, isotope pattern, and predicted elemental composition. The molecular relative exchange rate (in %) was calculated by averaging the peak area response for compounds containing ^13^C isotopes and normalizing it against the signal for present unlabeled analogs. The Relative Isotope Response was determined for annotated compounds. Based on the annotated elemental composition, accurate masses for all possible incorporations were predicted and chromatographic peak areas for extracted isotope ions were determined using Skyline. Peak areas were then normalized for each compound and sample, resulting in relative isotope responses (%) for each isotope incorporation state. The comparison to natural isotope abundances measured in unlabeled samples allows for the determination of ^13^C incorporations.

### Nucleus Isolation from Frozen Samples for snRNA Sequencing

Nucleus isolation of tissue samples was done as previously described.^[^ ^73^ ^]^ Briefly, tissue samples were cut into pieces <0.5 cm and homogenized using a glass Dounce tissue grinder (Sigma, cat. No. D8938). The tissue was homogenized 25 times with pestle A and 25 times with pestle B in 2 ml of ice‐cold nuclei EZ lysis buffer. The sample was then incubated on ice for 5 min, with an additional 3 mL of cold EZ lysis buffer. Nuclei were centrifuged at 500 g for 5 min at 4 °C, washed with 5 mL ice‐cold EZ lysis buffer, and incubated on ice for 5 min. After centrifugation, the nucleus pellet was washed with a 5 mL nuclei suspension buffer (NSB; consisting of 1 × PBS, 0.01% BSA, and 0.1% Rnase inhibitor (Clontech, cat. No. 2313A)). Isolated nuclei were resuspended in 2 ml NSB, filtered through a 35 µm cell strainer (Corning‐Falcon, cat. No. 352 235), and counted. A final concentration of 1000 nuclei per µL was used for loading on a 10x channel.

### snRNA‐seq Library Preparation and Sequencing

snRNA‐Seq libraries with Chromium Next GEM Single Cell 3ʹ Reagent Kits v3.1 on the Chromium Controller (10 × Genomics) were prepared. Single nucleus suspensions were prepared from cultured cell lines and nuclei were suspended in PBS containing 0.04% BSA. The nuclei suspension was loaded onto the Chromium Next GEM Chip G and ran the Chromium Controller to generate single‐cell gel beads in the emulsion (GEMs) according to the manufacturer's recommendation. Captured nuclei were lysed and the released RNA was barcoded through reverse transcription in individual GEMs. Barcoded, full‐length cDNA was generated and libraries were constructed according to the performer's protocol. The quality of libraries was assessed by Qubit 4.0 and the Agilent 2100. Sequencing was performed on the Illumina NovaSeq 6000 with a sequencing depth of at least 50 000 reads per nucleus and 150 bp (PE150) paired‐end reads (performed by Biomarker Technologies Corporation, Beijing, China).

### Pathogen Load

Peritoneal fluid was obtained by peritoneal lavage (7–8 mL in sterile PBS). Whole organs (i.e., liver, kidney, and spleen) were harvested and homogenized under sterile conditions by using a tissue homogenizer (Ultra Turrax T25 basic, IKA Labortechnik) at 15 000 min^−1^. Serial dilutions were plated onto Columbia Blood Agar with 5% Sheep Blood plates (ThermoFisher) and incubated (24 hr at 37 °C) in 5% CO2 (aerobes) or airtight container equipped with the OxoidTM AnaeroGenTM sachet (ThermoFisher) (anaerobes).

### Promethion Behavioral and Phenotyping System

Promethion Core (Sable Systems, USA) was used for indirect calorimetry experiments. Animals were singled cage and acclimatized for one day before experimental procedures were performed. The recording continued for one day after sham or CLP. The system consists of a standard GM‐500 cage with a food hopper and a water bottle connected to load cells (2 mg precision) with 1 Hz rate data collection. Additionally, the cage contains a red house enrichment. Total (ambulatory and fine) activity was monitored at 1 Hz rate using an XY beam break array (1 cm spacing). Oxygen, carbon dioxide, and water vapor were measured using a CGF unit (Sable Systems). This multiplexed system operated in pull‐mode. Airflow was measured and controlled by the CGF (Sable Systems) with a set flow rate of 2 L/min. Oxygen consumption and carbon dioxide production were reported in milliliters per minute (mL/min). Energy expenditure was calculated using the Weir equation^[^ ^74^ ^]^ and Respiratory Exchange Ratio (RER) was calculated as the ratio of VCO2/VO2. Raw data was processed using Macro Interpreter v2.38 (Sable Systems).

### Flow Cytometry

Single‐cell suspension from peritoneal lavage and bone marrow were obtained and immune cell population was quantified by flow cytometry. For bone marrow isolation, the femur was removed from mice after euthanasia, and ligament and muscles were mechanically cleared. The bone was separated in two pieces and bone marrow was collected by centrifugation in 1.5 mL microcentrifuge tubes. Peritoneal lavage was collected as indicated in Pathogen Load and washed with FACS buffer (2% BSA + 2 mM EDTA + 2 mM NaN3). Single‐cell suspension was stained in 5 mL polystyrene tubes with the following anti‐mouse antibodies to identify distinct immune cell populations: CD45 (clone REA737), CD3 (clone REA641), NK1.1 (clone REA1162), CD11b (clone REA592), Ly‐6G (clone REA526), F4/80 (clone REA126), and Ly‐6C (clone REA796) (Miltenyi Biotec) for 20 min at 4 °C. For absolute quantification, the CountBright Absolute Counting Beads (Invitrogen) kit was used according to the manufacturer´s instructions. Fluorescence minus one (FMO) control was used to identify positive populations in the analysis. Samples were acquired on an LSR II flow cytometer (BD Biosciences) and analyzed with FlowJo 10.7.1 software (TreeStar).

### Untargeted Metabolomics/Lipidomics Data Normalization

In both human and mice cohorts, metabolite data within annotation level 1, level 2a, and level 2b, and lipid data within annotation level 1, and level 2a were used. Metabolites and lipids were filtered out with Descriptive Power (DP) ≤ 2.5, missing percentage > 30% in all samples and replaced value < LOD (The Limit of Detection) with NA. Missing values in metabolome and lipidome profiles were imputed by LOD/2. In the human cohort, postoperative abundances of metabolites and lipids were normalized using the preoperative abundances to remove variability in the patient profiles that were unrelated to infection. Specifically, the post‐surgery data at three‐time points were log2 transformed, subtracted from the log2 transformed pre‐surgery data, and the mean of these differences was used. In the mice cohort, the data were logarithmically transformed before further analysis. Missing values in clinical profiles were imputed by the random forest method from R package missForest.^[^ ^75^ ^]^

### Partial Least‐Squares Discriminant Analysis (PLS‐DA)

Partial least‐squares discriminant analysis (PLS‐DA) was calculated by R package mixOmics.^[^ ^76^ ^]^ The ROC is calculated based on the first 2 predicted components to predict the class of each sample with auroc function from R package mixOmics.^[^ ^76^ ^]^ 95% confidence intervals of are under the curve (AUC) were calculated using the function roc from R package pROC (v1.18.5). Envfit function in the vegan package^[^ ^77^ ^]^ is used for vector fitting to fit the factors onto partial least‐squares discriminant analysis (PLS‐DA) ordination. Clinical indices were incorporated into the envfit function in the vegan package in R to determine those that were significant. The function envfit evaluates how each clinical variable individually correlates with axis1 and axis2. Metabolites and lipids were also analyzed against the community composition data using the envfit function in the vegan package in R. Statistical significance were assessed based on 999 permutations.

### Metabolic Features Identification in Human and Mice Cohort

In the human cohort, logistic regression models with sex, age, and organ dysfunction as covariates were built with R package stats.^[^ ^78^ ^]^ Metabolites and lipids were classified into subclasses by The Human Metabolome Database (HMDB)^[^ ^79^ ^]^ and Lipid Maps,^[^ ^80^ ^]^ respectively. Significant metabolites and lipids with raw P‐value ≤ 0.05 were shown in the color of the volcano plot. Ordinal logistic regression models with a cumulative logit link function were employed using the R package ordinal.^[^ ^81^ ^]^ Models were tested to meet the assumption of proportional odds. Benjamini‐Hochberg (BH) method was used to correct for multiple hypothesis testing. A list of 106 lipids and 15 metabolites were found to be significant (P ≤ 0.05 & BH P ≤ 0.25) in their respective ordinal regression models. The partial Spearman correlation was conducted by R package ppcor^[^ ^82^ ^]^ using significant metabolites and lipids (P‐value ≤ 0.05 and Benjamini–Hochberg P‐value ≤ 0.25) from ordinal regression models related to infection severity and clinical indices. Benjamini–Hochberg method was used to correct for multiple hypothesis testing.

In mice cohort, Student's t‐test was used to assess differences between the two groups for continuous variables. T statistics for lipids within the same class were compared against zero to identify significant up‐ or down‐regulated lipid classes, including only those with at least six lipids. One‐sided Fisher's exact test was used to identify lipid classes that significantly incorporated ^13^C‐labeled serine‐derived carbon. The Benjamini‐Hochberg procedure was applied to calculate the false discovery rate (FDR) and adjust p‐values for multiple hypothesis testing.

### Differential Correlation Analysis and Pathway Analysis

Differential correlations between the non‐infected and the infected patients were calculated using Spearman correlation with the R package DGCA.^[^ ^83^ ^]^ The significance of differential correlation was calculated for both same and different directions of the correlation coefficients between the two compared groups. The difference in Z‐scores, based on the correlation coefficients, was used to compute a two‐sided p‐value using the standard normal distribution. Empirical p‐values were calculated by comparing the Z‐scores from the original and permuted datasets. Only pairs with differential correlation (empirical p < 0.01) were included to build correlation networks from differentially correlated pairs. For sham and CLP mice, Pearson correlation was used to calculate differential correlations across five tissues. Only pairs with differential correlation (empirical p < 0.05) and at least one compound significantly correlated with the SOFA score in the human cohort were included. Multiscale embedded correlation networks were used to identify modules from the differential correlation network by R package MEGENA.^[^ ^84^ ^]^ Lipid pathway topology analysis was conducted by LION^[^ ^85^ ^]^ with “target‐list mode” by a one‐tailed Fisher's exact test. Lipids from the circled model in differential correlation analysis (Figure 1F) were used to form the list of compounds of interest. All lipids in our study were used as the background set.

### Integrative Analysis

Three data types (metabolomics, lipidomics, clinical indices) were obtained from experiments. The data consisted of 173 metabolites, 359 lipids, and 18 clinical indices. All data were Z‐scaled and concatenated into one matrix. Spearman correlations between all compounds were calculated by R package ppcor.^[^ ^82^ ^]^ Complete hierarchical clustering was used to reorder molecules based on 1 – Pearson correlation between all molecules by R package ComplexHeatmap.^[^ ^86^ ^]^ The number of clusters was determined based on the 5 clusters observed in the heatmap and cutree from R package dendextend.^[^ ^87^ ^]^ For each mega‐cluster, the over representation of the KEGG pathways was determined from the included metabolite by MetaboAnalyst 6.0^[^ ^88^ ^]^ and pathway enrichment analysis of the lipidomics data using the Lipid Ontology (LION) enrichment analysis web application.^[^ ^85^ ^]^ The SIRS+ group was constrained to the hierarchy that was established for the Sepsis group and yielded the same mega‐clusters for comparison.

### FEAST Analysis

To estimate the contribution of tissue source compounds to serum metabolite and lipid composition (“sink” communities), the fast expectation‐maximization (EM) microbial source tracking algorithm (FEAST R‐package v0.1.0) was used.^[^ ^34^ ^]^ The analysis was performed on scaled peak area tables, where peak areas were normalized by dividing each count matrix value by the maximum peak area and multiplying by 1 million, ensuring suitable scaling for integer conversion. The EM algorithm was run with 10 000 iterations.

### snRNA‐Seq Data Processing

Alignment was performed to this amended reference using 10x Cell Ranger v7.2, which employs the STAR sequence aligner. The reference genome was the human genome GRCm39 or the mouse genome mm39. Gene expression counts were determined using unique molecular identifiers (UMIs) for each cell barcode‐gene combination. Following alignment, cell barcodes were filtered to identify those that contain nuclei using the approach implemented in Cell Ranger, and only these barcodes were considered for downstream analysis. Cell Ranger count function was used to generate filtered matrices with “–force‐cell” was set as 10 000 for all liver, kidney, brain, and WAT tissues. To remove the nuclei with low quality, seurat (v5.1.0)^[^ ^89^ ^]^ R package was applied to construct 4 objects according to the four tissues and filter nuclei with gene number over 200 and below 5000, as well as the ratio of mitochondria lower than 10% were maintained, and genes with at least one feature count in more than ten nuclei were used for the following analysis. Doublets were identified performing the DoubletFinder (v2.0.4)^[^ ^90^ ^]^ R package with principal components (PCs) 1–20. nExp was set to 0.08 ×  nCells2/10000 and pN to 0.25. pK was determined through paramSweep_v3, and cells classified as doublets were removed before downstream analysis. In total, 43347, 48274, 49349, and 50929 cells were retained for liver, kidney, brain, and WAT, respectively. Next, Seurat's SCTransform function was applied to normalize and scale each sample, respectively. Replicates from the same tissue were integrated and removed batch effects using CCAIntegration method, and the top 50 PCs were used to conduct unsupervised clustering analysis, and subsequently visualized by t‐distributed stochastic neighbor embedding (tSNE) dimensionality reduction (resolution 0.8, 0.5, 0.5, and 0.3 for liver, kidney, brain, and WAT, respectively).

### Cell Type Annotation and Differentially Expressed Gene (DEG) Identification

Automatic and manual marker gene identification were conducted to annotate the cell types in different tissues. scTyper^[^ ^91^ ^]^ was first used to automatically annotate cell type of each cluster based on a combined reference database consisting of scType default database and cell type markers in CellSTAR database.^[^ ^92^ ^]^ Clusters with unknown labels were subsequently used for manual annotation. Seurat's PrepSCTFindMarkers function was applied at first, and FindAllMarkers function (avg_log2FC ≥ 0.5 and *p*_val_adj < 0.05) was used to find expressed markers of each cell cluster. Each unknown cluster was annotated on the basis of the expression level of typical gene markers for specific cell types. The detailed information of marker genes for each cell type across the four tissues is listed in Table S11 (Supporting Information). Seurat's FindMarkers function (avg_log2FC ≥ 0.5 and *p*_val_adj < 0.05) was used to identify the significantly different expressed genes between CLP and sham conditions in both tissue and cell type levels.

### Compass Analysis

For metabolic analysis, COMPASS was run with default parameters and the RECON2 model using the integrated SCTransform snRNA data as provided by Seurat's Cellranger pipeline (https://yoseflab.github.io/Compass/, –species parameter set to mus_musculus).^[^ ^93^ ^]^ The generated penalty reaction scores were subsequently log1p‐normalized for each single cell to receive probabilities of metabolic reaction activities per cell as described before.^[^ ^93^ ^]^ Reactions from “Glycine, serine and threonine metabolism”, “Lysine metabolism”, “Glycerophospholipid metabolism” and “Triacylglycerol synthesis” pathways were selected and further analyzed. Statistical significance (two‐sided wilcoxon test, FDR‐adjusted p ≤ 0.05) and effect size (Cohen's d measure of effect size) of change between CLP and sham mice were determined using the functions “wilcox_test” and “cohens_d” of the R package rstatix (v0.7.2, R version 4.3.0). To investigate identified metabolic reactions with most changes in CLP mice, these reactions were contextualized with information from the KEGG pathway maps hsa00310 (Lysine degradation), hsa00561 (Glycerolipid metabolism), hsa00564 (Glycerophospholipid metabolism) and hsa00260 (Glycine, serine and threonine metabolism, cf. Figure 4D).

### Energy‐Metabolism Related Cell–Cell Communication Identification and Gene Co‐Expression Network Construction

To investigate the variation of energy metabolism profiles in the four tissues, 34 energy pathways were selected belonging to mus musculus KEGG Orthology (KO) from the KEGG database Table S6 (Supporting Information), within which 13 genes specifically encoded by mitochondria were removed, leading to 1473 energy metabolic genes for downstream analysis. Principal component analysis (PCA) was performed based on average gene expression profiles of energy genes from the four different tissues. The four tissue‐specific Seurat objects were screened to four sub‐objects based on the 1473 energy metabolic genes. Cell‐cell signaling pathways in each cell type between CLP and sham groups across the four tissues were identified using the CellChat package (v2.1.2)^[^ ^39^ ^]^ in R based on the four sub‐objects. The meticulously curated database of ligand‐receptor (L‐R) interactions for mice was used for cell‐cell communication analysis, and the truncated mean was set to 5% to calculate the average energy metabolic gene expression by discarding 5% from each end of the data. CellChat takes snRNA‐Seq expression data and cell type annotations as input, and computes the number of each combination of sender and receiver cell types, as well as for each L‐R interaction.

Energy metabolic gene co‐expression networks of the four tissues were constructed using hdWGCNA (v0.3.3)^[^ ^94^ ^]^ R package. After filtering energy‐related metabolic genes with 5% prevalence across cells in each tissue, 1044, 1029, 957, and 973 genes were used for network construction for liver, kidney, brain, and WAT, respectively. Pseudobulk metacells aggregating nuclei were created that belonged to the same sample and cell type setting a nearest‐neighbour parameter k of 30 and using 10 as the maximum number of shared nuclei between metacells. Modules were identified through the ConstructNetwork function. To identify highly connected hub genes, ModuleConnectivity function was used to calculate the eigengene‐based connectivity (*kME*) of each energy metabolic gene. Ten genes were presented in each module with the highest *kME* value as hub genes, and used Cytoscape (v3.10.1)^[^ ^95^ ^]^ with edge‐weighted spring‐embedded layout to visualize the networks based on the topoligcal overlap matrices (TOMs) generated by GetTOM function in hdWGCNA.

### Statistical Analysis

R (version 4.3.0) and GraphPad Prism (version 10.3.1) were utilized for data analysis. Untargeted metabolomics/lipidomics data and snRNA‐Seq data were normalized by log2 transform and SCTransform function from R package Seurat respectively before downstream analyses. The values were presented as the means ± SDs. The data from the two unpaired groups were compared via a two‐tailed unpaired Student's t‐test. Statistical analyses requiring adjustment for confounding variables were performed using logistic regression and ordinal logistic regression models. Values of ^*^ *p* < 0.05, ^**^ *p* < 0.01, and ^***^ *p* < 0.001 were considered as statistically significant. The Benjamini‐Hochberg procedure was applied to calculate the FDR to adjust P values for multiple hypothesis testing.

## Conflict of Interest

M.S. received grants from NewB, Apollo Therapeutics, and UCL Technology Fund and others from Abbott, Amormed, bioMérieux, Biotest, Deltex Medical, Fresenius, Mindray, NewB, Pfizer, Radiometer, Roche Diagnostics, Safeguard Biosystems, Shionogi, and Spiden outside of this project. M.S. is an unpaid advisor to Presymptom Health Ltd, hemotune, deepUll, and Santersus, and R.L. is a part‐time advisor to Presymptom Health Ltd.

## Author Contributions

L.X. and Z.Z. contributed equally to this work. Conceptualization, G.P.; methodology, L.X., S.S., Z.Z.; Investigation, K.D., L.X., S.S., W.V., Z.Z.; writing—original draft, G.P., L.X., S.S., Z.Z.; writing—review & editing, all co‐authors; funding acquisition, G.P.; resources, G.P., M.B., M.S., R.L., S.W.; supervision, G.P.

## Supporting information



Supporting Information

Supporting Information

## Data Availability

Original data is available at the NIH Common Fund's National Metabolomics Data Repository (NMDR, supported by NIH grant U2C‐DK119886) website, the Metabolomics Workbench, https://www.metabolomicsworkbench.org where it has been assigned Project ID PR002122 (https://doi.org/10.21228/M8B52G). Raw snRNA sequencing data and feature, barcode, and matrix data processed by CellRanger without filtration are available from Gene Expression Omnibus (https://www.ncbi.nlm.nih.gov/geo/query/acc.cgi) with GEO accession IDs, GSE275689 (source: liver tissue), GSE275479 (source: brain tissue), GSE275740 (source: white adipose tissue), and GSE275526 (source: kidney tissue).
