# Supplementary material for: Multi‐Omics and ‐Organ Insights into Energy Metabolic Adaptations in Early Sepsis Onset
Source: Adv Sci (Weinh). 2025 May 24;12(30):e04418. doi: 10.1002/advs.202504418 (PMC12376650; doi:10.1002/advs.202504418)
Supplement: Supplementary file 1 — Supporting Information [file ADVS-12-e04418-s002.docx]

Supporting Information

**Multi-Omics and -Organ Insights into Energy Metabolic Adaptations in Early Sepsis Onset**

*Lin-Lin Xu, Zhengyuan Zhou, Sascha Schäuble, Wolfgang Vivas, Karen Dlubatz, Michael Bauer, Sebastian Weis, Mervyn Singer, Roman Lukaszewski, Gianni Panagiotou**

**Figure S1
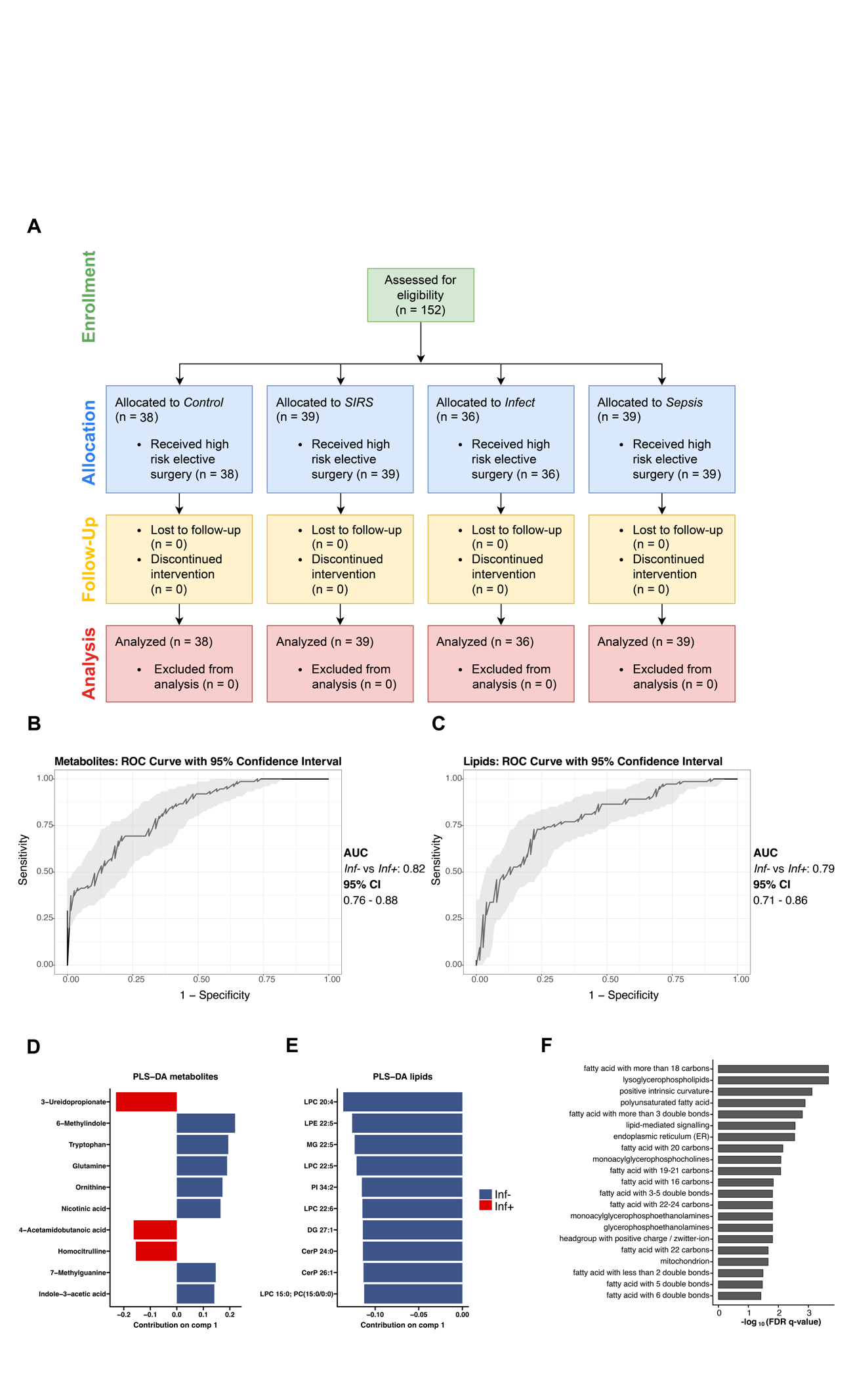
**

**Figure S1. Supplementary figure of Infected versus Non-Infected patients (related to Figure 1).**

(A) CONSORT diagram of the studied cohort. n: number of patients. (B, C) ROC curve (receiver operating characteristic curve) of PLS-DA in metabolites and lipids. (D, E) Top 10 important features selected by PLS-DA in metabolites and lipids. (F) Enriched lipid pathway from the selected model of Differential Correlation Analyses (associated to Figure 1E). Members from the circled module were used for pathway analyses with Lipid Ontology (LION) enrichment analysis web application. LION terms with FDR P-value ≤ 0.05 were presented.

**Figure S2
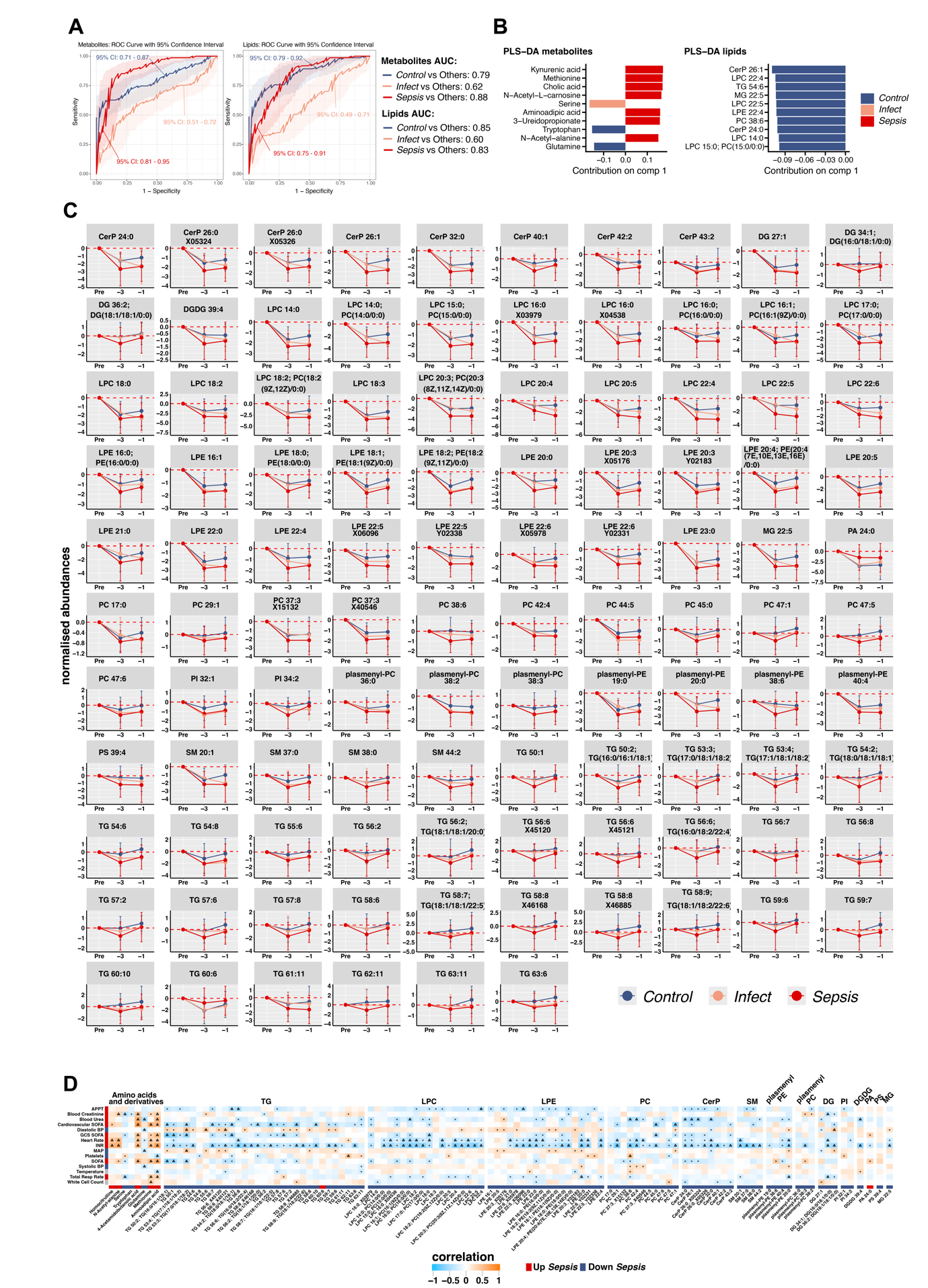
**

**Figure S2. Supplementary figure related to Infection severity (related to Figure 2).**

(A) ROC curve (receiver operating characteristic curve) of PLS-DA in metabolites and lipids. (B) Top 10 important features selected by PLS-DA in metabolites and lipids. (C) Significant lipids in ordinal regression (related to Figure 2D). Individual abundance changes of lipids selected by ordinal regressions. (D) Partial Spearman correlation network of significantly altered compounds from Figures 2C and 2D and clinical indices in Sepsis group. Correlations with raw p ≤ 0.05 are indicated with a circle and FDR ≤ 0.05 are indicated with a triangle. Negative correlations are shown in blue and positive correlations are shown in yellow. The Color bar indicates increasing (red) and decreasing (blue) trends with infection severity of each variable tested by ordinal regression models (compounds) and literature review (clinical data).

**Figure S3**

**Figure S3. Integrative Correlation Analysis Reveals Different Metabolic Programs of Sepsis and SIRS.**

Spearman correlation matrixes (covariates: age and sex) of a total of 550 observations, including 18 clinical variables (black), 173 metabolomics (green) and 359 lipidomic (blue) in Sepsis (lower triangle matrix) and SIRS (upper triangle matrix) group. Hierarchical clustering identified 5 mega-clusters (outlined in black; Table S4). clinical variables and pathways of interest in each cluster are highlighted.

**Figure S4
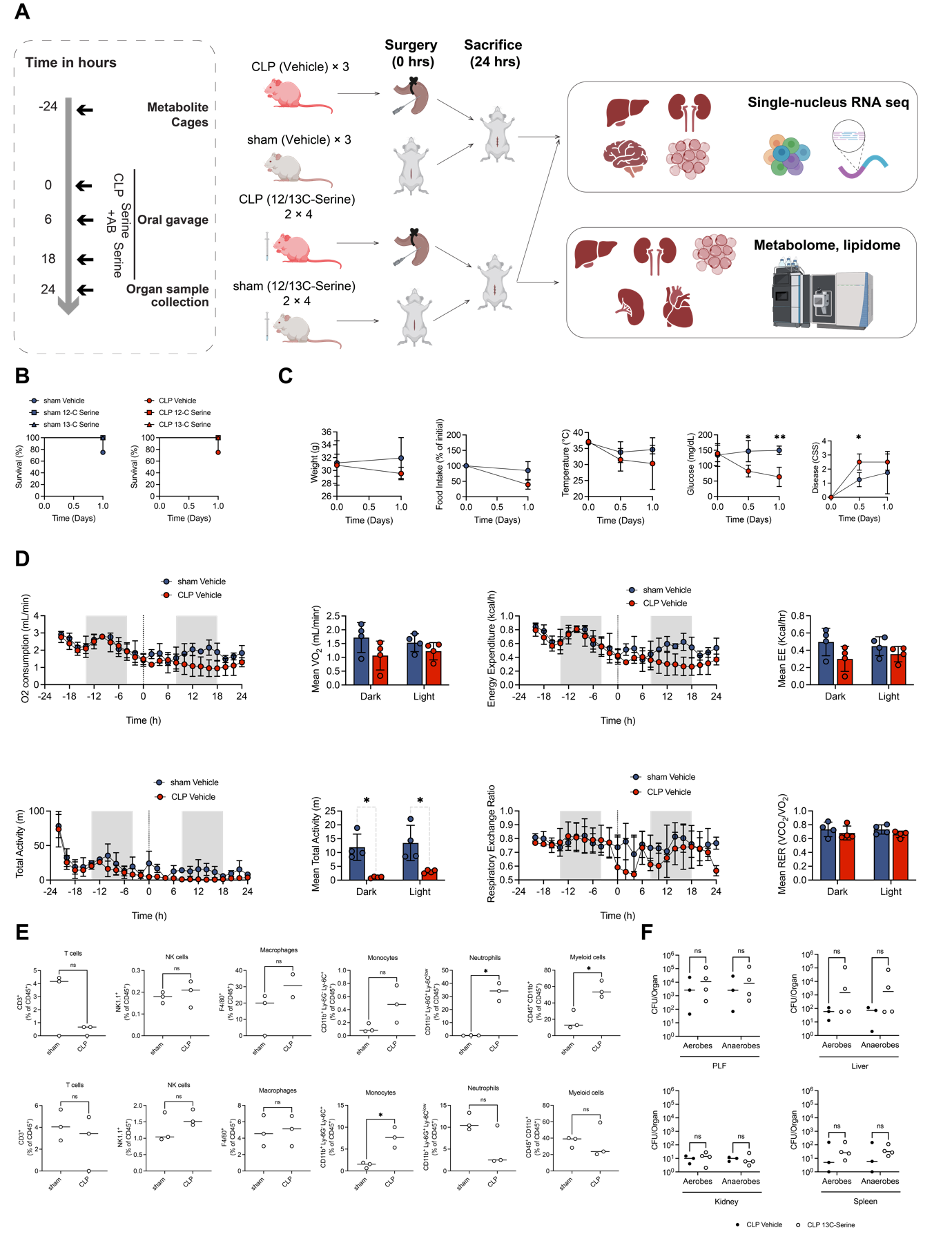
**

**Figure S4. In vivo mice model.**

(A) Experimental design of the in vivo mouse model of sepsis. Numbers behind CLP and sham indicate number of mice per condition and experiment. AB: antibiotics. Created with Biorender.com. (B) Survival of CLP and sham mice (four mice per group). (C) Body weight, food intake, temperature, glucose, and disease score for both CLP and sham mice at day 0, 0.5 (12h), and 1 (24h). (D) Activity monitoring every 2 hours of CLP and sham mice. Barplots indicate significance of change (two-tailed Student's t-test) (E) Immune phenotyping of CLP and sham mice from primary lymphoid follicle (first row) and bone marrow (second row). Significance evaluated by two-tailed Student's t-test. (F) Pathogen load in septic mice. Significance evaluated by two-tailed Student's t-test. *p ≤ 0.05; **p ≤ 0.01; ***p ≤ 0.001.

**Figure S5
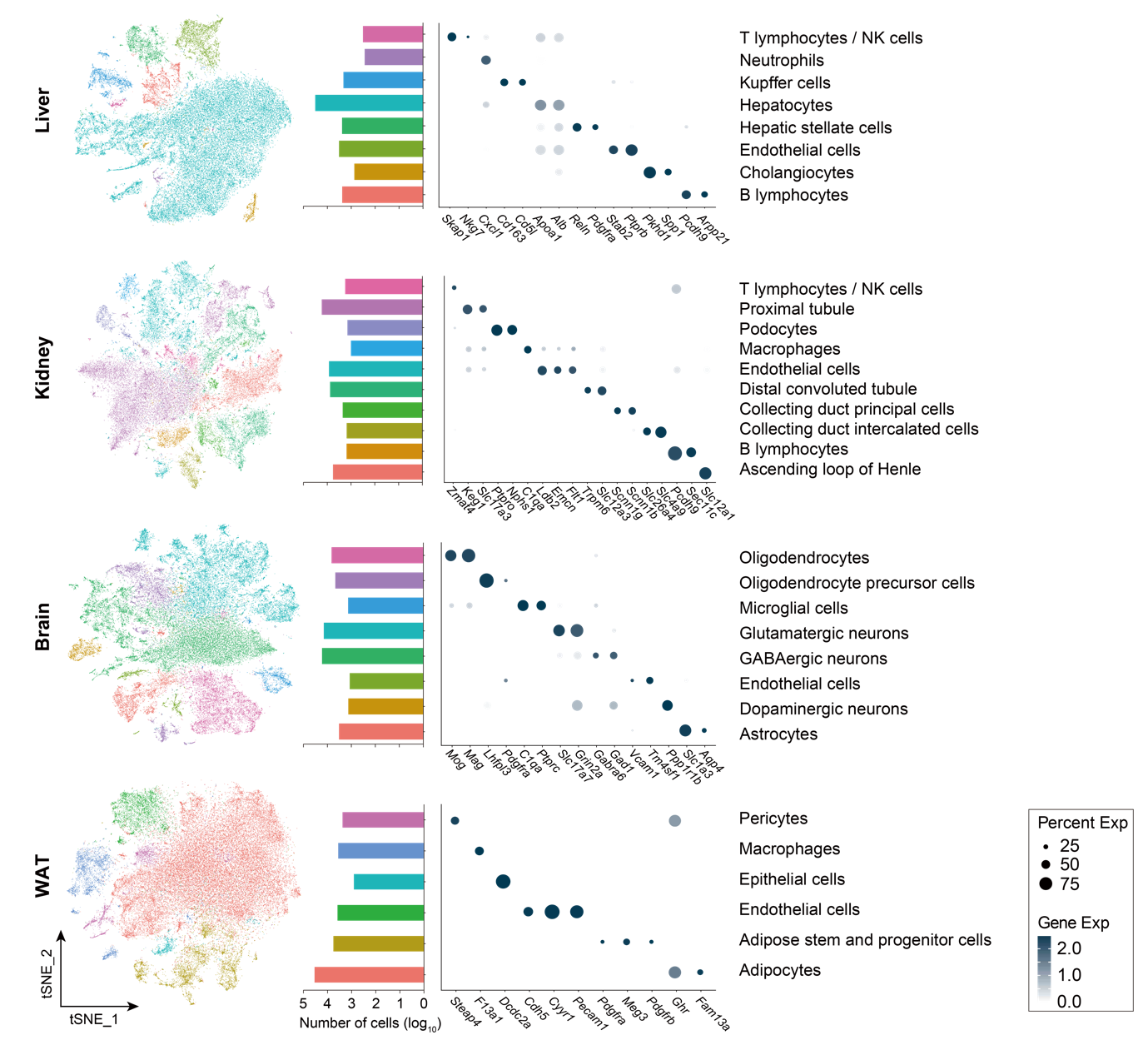
**

**Figure S5. Cell type annotation based on marker genes identified through snRNA sequencing in liver, kidney, brain, and WAT of mice models.**

t-SNE plots represent the cell cluster profiles of the four tissues in mice models with different colors referring to different cell types (left plots). Bar plots represent the numbers of cells in different cell types in the four tissues with log10 transformation (middle panels, colors matched with t-SNE plots per tissue). Dot plots represent the identified marker genes to indicate cell types per organ tissue (right panels).

**Figure S6
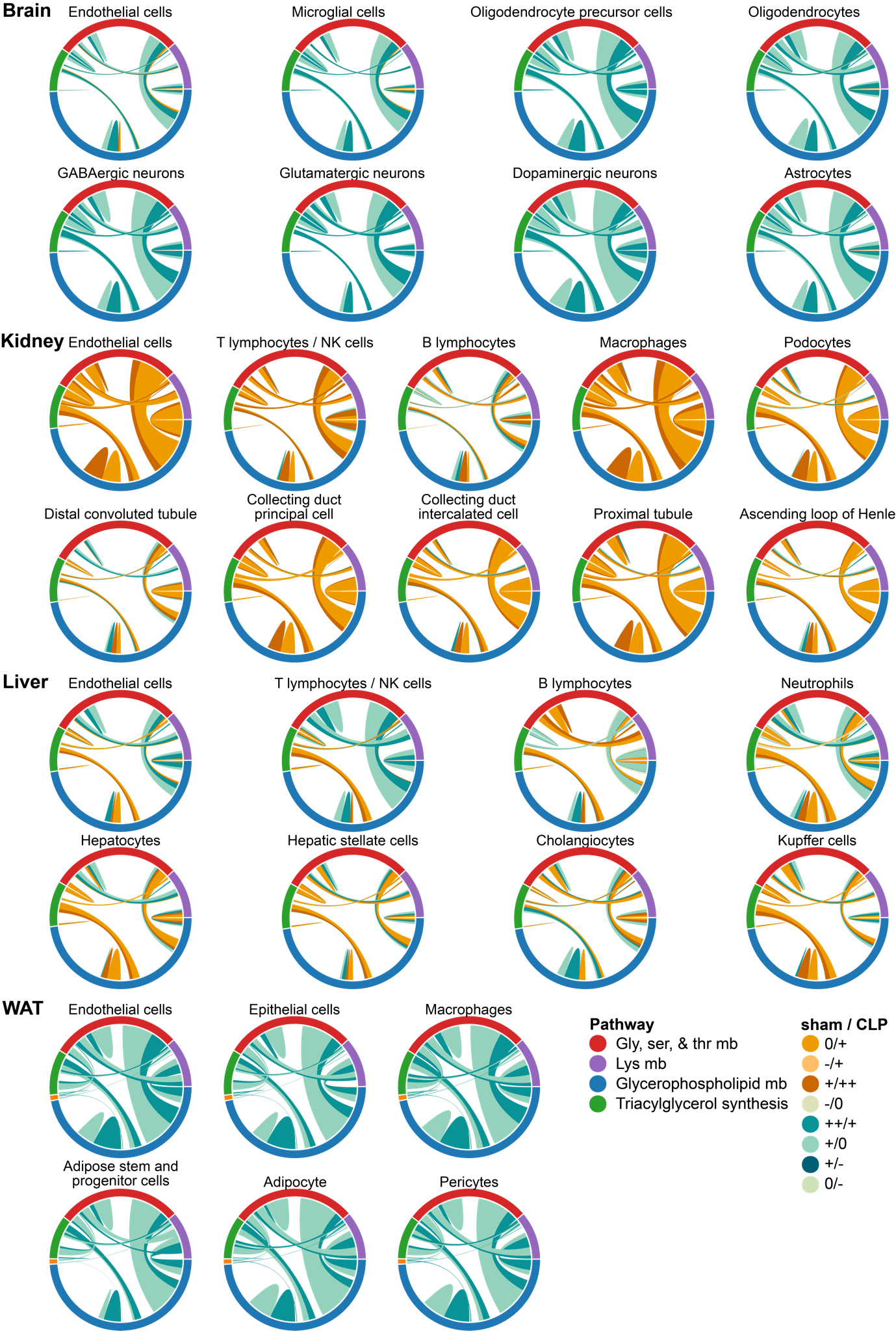
**

**Figure S6. Spearman correlations of Compass-based metabolic reaction probabilities per cell type and tissue.**

Circos chord charts displaying spearman correlations between all reactions per cell type (paired by snRNA associated barcode per cell). Abbreviations: Gly, ser, ala & thr mb: Glycine, serine and threonine metabolism; Lys mb: Lysine metabolism, Glycerophospholipid mb: Glycerophospholipid metabolism.

**Figure S7
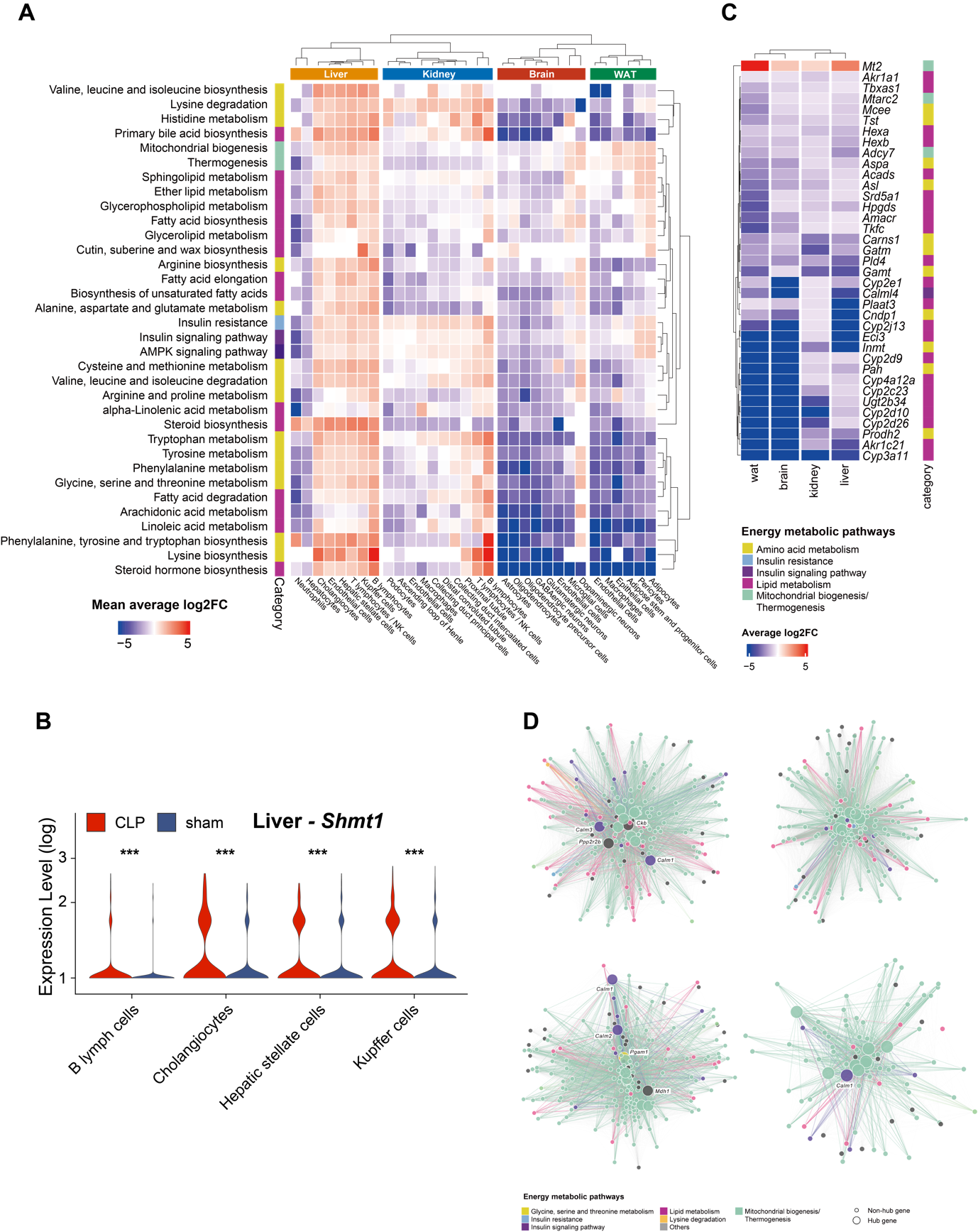
**

**Figure S7. Energy metabolism alterations based on snRNA sequencing in mice models.**

(A) Profile of 34 energy metabolic pathways at the cell type level based on the mean average log2(fold-change) of genes belonging to each pathway between CLP and sham. Red and blue colors indicate up- and down-regulated genes in CLP, respectively. (B) Gene expression levels of Shmt1 in liver B lymphocytes, Cholangiocytes, hepatic stellate cells, and Kupffer cells. (C) Profile of the 37 DEGs with consistent expression changes at tissue level based on the average log2(fold-change) of genes in the four tissues between CLP and sham. (D) Co-expression networks based on energy metabolism associated genes in the common module per tissue. Each node represents one gene and each edge refers to the co-expression relationship between two connected nodes. Node color reflects pathway association. Nodes of hub genes not belonging to mitochondrial biogenesis/thermogenesis are enlarged and labelled with gene symbols. Colored linkages indicate the pathway origin of genes linked with the hub genes.

**Supplemental Tables**

**Table S1.** Patient parameters and sampling information.

**Table S2.** All metabolites and lipids signatures identified in human and mice.

**Table S3.** Six lipid modules were identified from the global networks via multiscale clustering analysis.

**Table S4.** Five mega-clusters obtained by hierarchical clustering. (related to Figure S3).

**Table S5.** Cohen’s d effect size of top5 negatively and positively changed COMPASS-derived metabolic reaction probability scores. Related to Figure 4C and 4D.

**Table S6.** Information of 1,473 genes referring to 34 energy-related metabolic pathways from the KEGG database.

**Table S7.** All co-expressed genes belonging to the common module related to mitochondrial biogenesis / thermogenesis in each tissue.

**Table S8.** Student's t test of administration of serine to sham-operated and CLP mice.

**Table S9.** Incorporation of 13C-labelled serine to sham-operated and CLP mice.

**Table S10.** External cohorts for signature metabolites and lipids.

**Table S11.** Information of marker genes used for cell type annotation.
